# Beneficial Effects of Traditional Fermented Soybean Sauce (Kanjang) on Memory Function, Body Water, and Glucose Metabolism: Roles of Gut Microbiota and Neuroinflammation

**DOI:** 10.3390/nu17101617

**Published:** 2025-05-08

**Authors:** Yu Yue, Hee-Jong Yang, Chen Li, Myeong-Seon Ryu, Ji-Won Seo, Do Youn Jeong, Sunmin Park

**Affiliations:** 1 Department of Bioconvergence, Hoseo University, Asan 31499, Republic of Korea; yuyue6491@gmail.com (Y.Y.); lic77732@gmail.com (C.L.); 2 Department of R & D, Microbial Institute for Fermentation Industry, Sunchang 56048, Republic of Korea; godfiltss@naver.com (H.-J.Y.); rms6223@naver.com (M.-S.R.); wldnjs8769@naver.com (J.-W.S.); 3 Department of Food and Nutrition, Obesity/Diabetes Research Center, Hoseo University, Asan 31499, Republic of Korea

**Keywords:** soy sauce, memory impairment, gut microbiota, neurotrophic signaling, gut microbiota

## Abstract

**Background:** Traditional fermented soybean foods, acting as potential synbiotics, may help mitigate cognitive impairment associated with amnesia. This study investigated the neuroprotective effects of four kanjang (Korean fermented soy sauce) varieties and their underlying mechanisms. **Methods:** Male Sprague Dawley rats (n = 70) were divided into seven groups: normal control, scopolamine control, positive control (1 mg/kg bw/day of donepezil), and four scopolamine-treated groups receiving different kanjang varieties (0.5% in high-fat diet). Based on their *Bacillus* content, the kanjang samples were categorized as traditionally made kanjang (TMK) with high *Bacillus* (SS-HB), TMK with medium *Bacillus* (SS-MB), TMK with low *Bacillus* (SS-LB), and factory-made kanjang (SS-FM). **Results:** Scopolamine administration disrupted energy, glucose, and water metabolism and impaired memory function (*p* < 0.05). All kanjang treatments improved insulin sensitivity, reduced inflammation, enhanced glucose tolerance, and decreased visceral fat. SS-MB, SS-HB, and SS-FM increased skeletal muscle mass. They maintained body water homeostasis by suppressing the renin–angiotensin–aldosterone system. Kanjang treatments improved memory function, with SS-FM showing the least significant effects. The treatments reduced neuronal cell death in the hippocampal CA1 region, decreased acetylcholinesterase activity, and increased brain-derived neurotrophic factor mRNA expression. Gut microbiota analysis revealed that kanjang treatments increased Lactobacillaceae and decreased Lachnospiraceae, with SS-HB and SS-LB specifically elevating *Ligilactobacillus*. Metagenomic analysis demonstrated enhanced glycolysis/gluconeogenesis pathways and enhanced butanoate metabolism while reducing lipopolysaccharide biosynthesis and pro-inflammatory signaling. SS-MB and SS-LB increased intestinal goblet cell counts and the serum butyrate concentration. **Conclusions:** These findings suggest that kanjang consumption, particularly SS-HB and SS-LB varieties, can ameliorate memory impairment in this murine model through multiple mechanisms: metabolic improvements, enhanced neurotrophic signaling, gut microbiota modulation, and reduced neuroinflammation via gut–brain axis activation. Human clinical trials are warranted to determine if these promising neuroprotective effects translate to clinical applications.

## 1. Introduction

Kanjang, a traditional Korean fermented soy sauce, has historically served as a primary source of sodium in Asian cuisine, particularly in Korea [1]. The fermentation process generates bioactive compounds, including peptides, amino acids, and phenolic compounds, a characteristic distinguishing its physiological effects from those of regular salt. In Asian cultures, mainly where rice is a staple food, fermented soybeans have traditionally been used not only as flavor enhancers but also as nutrient supplements [1]. The preservation process necessitates using salt in fermentation, leading to the development of various fermented soybean products, with kanjang emerging as a globally recognized seasoning.

Memory deficit disorders represent a growing global health concern, particularly with an increasing aging population and the rising prevalence of neurodegenerative diseases. Dietary factors play a crucial role in cognitive function, with salt intake emerging as a significant modulator. Despite the general association between high salt intake and adverse health outcomes [2], traditional fermented soybean products (jang) show inverse associations with components of the metabolic syndrome, including abdominal obesity, hypertension, dyslipidemia, and hyperglycemia, as demonstrated by the Korean Genome and Epidemiology Study (KoGES) involving a large cohort of 58,701 participants [3]. Fermented soybean consumption has been shown to reduce the sodium/potassium ratio and serum aldosterone concentrations, thereby decreasing water retention and inflammation in estrogen-deficient animal models [4]. Furthermore, these products modify the gut microbiota composition and improve intestinal permeability, establishing a potential mechanism through the gut–brain axis for improving cognitive function [5]. The composition and metabolites of the gut microbiota significantly influence cognitive performance through the production of various neuroactive metabolites acting on the gut–brain axis and affecting brain function directly or indirectly through immune and endocrine pathways [6]. Herbal extracts and probiotics have been shown to modulate gut microbiota and metagenome function, thereby enhancing cognitive function [7,8].

The present study employed male Sprague Dawley rats with scopolamine-induced memory impairment as an established model to investigate the effects and mechanisms of kanjang (traditional Korean soy sauce) on cognitive function. The scopolamine-induced amnesia model was selected for its reproducibility and translational value in studying memory impairment, as it mirrors the aspects of cholinergic dysfunction observed in human cognitive disorders [9]. This model enables the systematic examination of how different soy sauce varieties affect memory function, providing insights relevant to age-related cognitive decline in humans. The objectives of this study were as follows: (1) evaluate the effects of four different kanjang varieties on memory function in scopolamine-induced amnesic rats; (2) analyze changes in the gut microbiota composition and metagenomic functional profiles in response to different kanjang treatments; (3) investigate the relationship between gut microbiota changes, water metabolism, and cognitive performance; and (4) elucidate the underlying mechanisms by which kanjang-induced alterations in the gut microbiota influence memory function. This approach leverages the similarities between rat and human gut microbiota compositions and the conserved gut–brain axis pathways, enhancing the potential applicability of our findings to human cognitive health. Understanding these mechanisms could provide valuable insights into developing more beneficial sodium sources for improving cognitive health based on their impact on the gut microbiota and overall body physiology.

## 2. Materials and Methods

### 2.1. Kanjang Production and Sample Collection

In Korea, soy sauce is traditionally produced using two main fermentation methods: traditional fermentation (TMK) and microorganism-inoculated fermentation. TMK was produced through three sequential fermentation steps: (1) the preparation of meju (fermentation of crushed, boiled soybeans with rice straw at 20–25 °C for 40–50 days); (2) fermentation in brine (meju was soaked in 16–18% salt solution outdoors for 40–60 days); and (3) secondary fermentation (separation of liquid TMK and solid doenjang fermented for >6 months). TMK samples were collected from multiple Korean provinces to capture regional differences in bacterial communities and metabolite profiles. Initial analyses focused on measuring sodium content, identifying bacterial composition, and quantifying biogenic amine levels. For comparison, factory-made kanjang (FMK), produced through a shortened fermentation process involving inoculation with *Aspergillus* spp. and *Bacillus* spp., was also analyzed. The soy sauce (TMK) samples were classified into three categories based on *Bacillus* abundance: (1) high *Bacillus* (SS-HB), (2) medium *Bacillus* (SS-MB), and (3) low *Bacillus* (SS-LB). An additional sample type, factory-made kanjang from Sunchang (Jeollabuk-do, Republic of Korea), was labeled SS-FM.

### 2.2. Sodium, Aflatoxin, and Biogenic Amine Analyses of Kanjang Samples

The sodium content in the kanjang samples was measured using inductively coupled plasma atomic emission spectroscopy (ICP-AES, Thermo IRIS Intrepid II XDL, Santa Clara, CA, USA) after nitric acid digestion, with argon flow at 10.00 L/min and detection at 589.0 nm. For aflatoxin analysis, TMK was extracted with 70% methanol (1% NaCl), filtered, passed through an Afla test column, and analyzed by high-performance liquid chromatography (HPLC, Agilent 1200, Agilent Technology, Santa Clara, CA, USA) with an Ultra Grade 120 column (Shiseido Co., Osaka, Japan) using a acetonitrile/water (25:75) mobile phase at 1.0 mL/min, detected using a fluorescence detector (FLD) (ex: 360 nm; em: 450 nm).

The biogenic amine contents in the kanjang samples were derivatized with 1,7-diaminoheptane (internal standard), saturated Na_2_CO_3_, and 1% dansyl chloride. Histamine and tyramine standards (0.1–100 mg/L) were prepared in 0.01 N HCl. After ethyl ether extraction and evaporation, the residues were dissolved in acetonitrile for the HPLC analysis (Agilent 1200) using a Capcell Pak C18 column (Agilent 1200).

### 2.3. Animal Care

Seventy male 8-week-old Sprague Dawley (SD) rats (192 ± 7.4 g) were obtained from Daehan Bio Inc. (Eum-Sung, Republic of Korea) and acclimated for one week at the animal facility at Hoseo University. Each rat was housed individually in a polycarbonate cage under controlled conditions (23 °C, 12 h light/dark cycle) with ad libitum access to food and water. This study complied with the instructions provided in the Guide for the Care and Use of Laboratory Animals (8th edition, National Institutes of Health [NIH], US) and was approved by the Hoseo University Institutional Animal Care and Use Committee (HSUIACUC-23-034, May 23, 2023).

### 2.4. Induction of Memory Deficit in an Animal Model

Memory impairment was induced by daily intraperitoneal (IP) injections of scopolamine (2 mg/kg body weight) for 8 weeks. Scopolamine administration suppresses the parasympathetic nervous system (PNS), leading to cognitive deficits that model memory impairment. In the positive control group, donepezil (1 mg/kg body weight) was administered daily via oral gavage immediately after the scopolamine injection. The normal control group received daily IP injections of physiological saline (0.9% NaCl) of a volume equivalent to scopolamine.

### 2.5. Experimental Design and Diet Preparation

Sample size calculations were conducted using the G*Power 3.1 software (https://www.psychologie.hhu.de/arbeitsgruppen/allgemeine-psychologie-und-arbeitspsychologie/gpower) with power = 0.85 and effect size = 0.50, resulting in 10 rats per group to assess the main effects. No specific inclusion or exclusion criteria for animals or data points were established a priori. All animals initially enrolled in this study completed the experimental procedures without complications. Consequently, no animals or data points were excluded at any stage of the experiment or analysis. Each experimental group consisted of 10 rats (n = 10), and all animals were included in the final statistical analysis.

Male Sprague Dawley rats were randomly assigned to seven groups (n = 10 per group): normal control (high-fat diet [HFD] + saline), control (HFD + scopolamine), positive control (HFD + scopolamine + donepezil), SS-HB, SS-MB, and SS-LB (HFD + 0.5% of the respective TMK extract [HB, MB, or LB] + scopolamine), and SS-FM (HFD + 0.5% SS-FM + scopolamine) (Figure S1). Randomization was conducted using a computer-generated random sequence to ensure unbiased group allocation. To minimize potential confounding factors, animals were housed individually, and cage positions were rotated weekly. Behavioral testing and sample collection were carried out in a randomized order. Blinding was applied during outcome assessment and data analysis, with assessors unaware of group assignments. Each diet formulation was labeled with a coded number, and the animal care staff were blinded to the contents and treatment allocation of each diet.

All the diets were based on a modified polyphenol-free AIN-93 HFD containing 37% of carbohydrates, 20% of protein, 43% of lard, and essential micronutrients. This polyphenol-free diet was specifically chosen to establish a controlled baseline without dietary polyphenol interference, as TMK’s potential cognitive benefits are potentially attributed to its polyphenol content. The treatment diets were supplemented with a 0.5% freeze-dried kanjang sample of a specific variety with cornstarch, casein, corn oil, and salts (NaCl) adjusted to maintain the equivalent nutrient composition (43% energy from fat, 4.65 kcal/g). The diets were stored at 4 °C until use, and rats had ad libitum access to food and water throughout the study. Food efficiency was calculated by dividing weekly food intake by weight gain in the last week of the intervention.

### 2.6. Oral Glucose Tolerance Test (OGTT) and Intraperitoneal Insulin Tolerance Test (IPITT)

During the 5th week of the dietary intervention, rats were fasted for 16 h overnight, followed by the oral administration of glucose at a dose of 2 g/kg body weight for OGTT. Blood samples were collected from the tail vein at 0, 15, 30, 60, and 120 min after glucose administration to measure serum glucose concentrations. In addition, serum insulin concentrations were assessed at 0, 20, 40, and 90 min to evaluate the insulin response. After completion of the OGTT, animals were returned to their normal feeding. On the following day, after a 6 h fasting period, the IPITT was conducted. Rats received an intraperitoneal insulin injection at a dose of 1 U/kg body weight, and blood samples were collected from the tail vein at 0, 15, 30, 60, and 120 min post-injection to assess serum glucose concentrations and determine insulin sensitivity.

### 2.7. Assessments of Memory Deficit

Memory assessments were conducted using passive avoidance, Y-maze, novel object recognition, and the Morris water maze tests at the 5–6-week intervention (Figure S1). In the passive avoidance test, the rats were placed in a light–dark shuttle box 50 min post-scopolamine injection on the day of the memory function assessment. During the acquisition trials, the rats received an electric stimulus (75 V, 0.2 mA, 50 Hz) upon entering the dark chamber. After 16 h, the latency to enter the dark chamber was recorded (cutoff: 600 s).

The Y-maze test measured spontaneous alternation behavior. The rats were placed in a Y-shaped maze (50.5 cm × 20 cm × 20 cm) for 8 min. Correct alternation was defined as sequential entry into all three arms, and the alternation percentage was calculated as correct alternations divided by total arm entries.

In the novel object recognition test, after three days of acclimation, the rats explored two identical objects for 10 min (training phase). One hour later, one object was replaced with a novel object, and exploration was recorded for 5 min. The recognition index was calculated as the time spent exploring new versus familiar objects.

Finally, the Morris water maze test evaluated hippocampal-dependent spatial memory. The rats were placed in zone 1 of a water-filled pool and trained to find a platform placed in zone 5. Training occurred over days 1, 2, and 3, and on Day 5, the rats were tested to find zone 5 without a platform. The latency time, frequency of visits, and duration spent in zone 5 were recorded with a 600 s cutoff.

### 2.8. Sample Collection and Biochemical Assays

After the Morris water maze test, the rats underwent 16 h of fasting before sample collection. Blood was obtained from the portal vein and inferior vena cava following ketamine–xylazine anesthesia. The epididymal and retroperitoneal fat masses were measured. The hippocampi from six randomly selected rats per group were dissected, divided, and frozen at −70 °C. The remaining four rats had their brains preserved in a 20% sucrose solution at 4 °C overnight, then frozen at −20 °C. The large intestine tissues were washed with phosphate-buffered saline (PBS) and fixed in 4% paraformaldehyde (pH 7.2). The serum was separated from the portal vein blood, and the fecal samples from the cecum were stored at −70 °C. The serum tumor necrosis factor-alpha (TNF-α) and interleukin-1 beta (IL-1β) were quantified using ELISA kits (eBioscience, Santa Clara, CA, USA).

One portion of the hippocampus was lysed in a radioimmunoprecipitation assay (RIPA) buffer, and the supernatants were used to measure triglycerides, cholesterol, and lipid peroxide levels via spectrophotometric kits (DoGenBio, Seoul, Republic of Korea). These supernatants were digested with α-amyloglucosidase (Sigma) to assess the glycogen levels using a glucose spectrophotometric kit (DoGenBio). Acetylcholinesterase (AChE) activity was measured using a rodent AChE ELISA kit (Elabscience, Houston, TX, USA). The remaining hippocampal portion was processed with a Trizol reagent (Invitrogen, Carlsbad, CA, USA) for total RNA extraction.

Serum aldosterone, renin, and angiotensin II concentrations and angiotensin-converting enzyme activity were measured using ELISA kits (eBioscience) for water metabolism.

### 2.9. Quantitative Real-Time PCR to Evaluate mRNA Expression in the Hippocampal Tissue

cDNA synthesis was performed by combining isolated total RNA with Superscript III reverse transcriptase and high-fidelity Taq DNA polymerase (1:1:1, *v*:*v*:*v*). The synthesized cDNA was mixed with target gene primers and SYBR Green mix for real-time quantitative PCR analysis (BioRad, Hercules, CA, USA). Target genes included a ciliary neurotrophic factor (*CNTF*), brain-derived neurotrophic factor (*BDNF*), *TNF-α*, *IL-1β*, and β-actin. Gene expression levels were analyzed using the comparative cycle threshold (CT) method [10].

### 2.10. Histological Analysis of the Brain and Large Intestinal Tissues

The brain was further equilibrated in a 20% sucrose solution in PBS at 4 °C for 48 h and then frozen at −20 °C. Using a cryostat microtome, the frozen brain was sectioned into 10 µm thick coronal slices, which were stained with a crystal violet solution to assess damaged cells in the hippocampal region under a microscope.

For the large intestinal tissue analysis, two serial 5 μm paraffin-embedded sections were taken from the seventh or eighth sections to avoid duplicate site analysis. The sections were stained with hematoxylin–eosin (H-E) and Alcian blue–perchloric acid–Schiff (AB-PAS) staining. Impaired cells in the H-E-stained sections were counted using a Zeiss Axiovert 5 microscope (Zeiss, Oberkochen, Germany) with the Dilation X-ray Imager (DIXI) imaging solution and scored from 0 to 5, based on the relative area of damaged cells. The percentage of goblet cells producing mucin was determined from the AB-PAS-stained sections, and their proportion within the intestinal area was calculated.

### 2.11. Assessment of Serum Short-Chain Fatty Acid (SCFA) Concentrations

Serum samples were treated with ethanol (Duksan, Republic of Korea), followed by 1N hydrochloric acid (HCl) (100:1 ratio). After vortexing, the mixture was centrifuged at 15,000 rpm for 15 min at 4 °C. The SCFA concentrations were determined using a gas chromatograph (GC, Clarus 680 GAS, PerkinElmer, Waltham, MA, USA) equipped with an Elite-free fatty acid phase (FFAP) capillary column (30 m × 0.25 mm × 0.25 μm) [11]. External standards for acetate, propionate, and butyrate (Sigma Co., St. Louis, MO, USA) were used for quantification.

### 2.12. Gut Microbiome Composition and Metagenome Analysis

The bacterial DNA from the fecal samples was extracted using the Power Water DNA Isolation Kit (Qiagen, Hilden, Germany). DNA was amplified using 16S amplicon primers, and libraries were prepared following the GS FLX plus protocol. Sequencing was performed on the Illumina MiSeq system (Genome Sequencer FLX plus; Roche Co., Basel, Switzerland) at Macrogen, Seoul. The 16S amplicon sequences were processed using Mothur v.1.36 and aligned with the Silva reference alignment v.12350 for determining bacterial taxonomy.

Taxonomic assignments and bacterial counts were analyzed using principal coordinates analysis (PCoA) in the R package. Linear discriminant analysis effect size (LEfSe) analysis identified primary bacteria in each group at the species level. Functional metabolic predictions were performed using the Phylogenetic Investigation of Communities by Reconstruction of Unobserved States (PICRUSt)2, with predicted functions mapped against the Kyoto Encyclopedia of Genes and Genomes (KEGG) Orthology database. The Sparse Correlations for Compositional Data (SparCC) algorithm was used to examine microbial species relationships, excluding taxa showing no significant differences between groups or having correlation coefficients below 0.1. The species co-occurrence network was visualized using Cytoscape version 3.4.0.

### 2.13. Statistical Analyses

Statistical analyses were performed using SAS version 7 (SAS Institute, Cary, NC, USA). Data were presented as means ± standard deviations (SDs). Normally distributed variables were analyzed using univariate analysis, and group comparisons were performed using one-way analysis of variance (ANOVA). When significant intergroup differences were observed in ANOVA, post hoc Tukey tests were conducted for multiple comparisons. A *p*-value < 0.05 was considered statistically significant.

## 3. Results

### 3.1. Characteristics of the Kanjang Samples

Four kanjang samples with varying microbial compositions, sodium content, and biogenic amine levels were selected for analysis. The samples showed distinct profiles: SS-HB predominantly contained *Bacillaceae* (82%), had high sodium content (8.59%), and moderate levels of viable bacteria (1.3 × 10^5^ colony-forming units [CFU]) (Table S1, Figure S2). SS-MB was characterized by high levels of *Enterobacteriaceae* (88%), moderate levels of *Bacillaceae* (17%), lower sodium content (5.56%), and high concentrations of viable bacteria (1.0 × 10^7^ CFU). SS-LB featured *Enterobacteriaceae* (20%), low *Bacillaceae* (4%), and Enterococcaceae (25%) (Table S1, Figure S2). This sample had the highest biogenic amine content (histamine and tyramine), high sodium levels (9.39%), and viable bacteria of 4.3 × 10^5^. SS-FM exhibited high levels of *Enterobacteriaceae* (88%), moderate *Bacillaceae* (17%), and *Halomonadaceae* (25%), with medium sodium content (6.08%) and low viable bacterial counts (1.0 × 10^3^ CFU) (Table S1, Figure S2). These varying microbial and chemical compositions influenced the metabolite profiles of each kanjang sample, potentially affecting their impact on memory impairment.

### 3.2. Effects of Kanjang Treatments on Weight Gain

The administration of the scopolamine injection to the control group disrupted energy metabolism compared to the normal control group without the scopolamine injection. The control group exhibited a higher final body weight and weight gain during the experimental period than the normal control group, though no significant differences were observed between the control and positive control groups (Table 1). All treatments with the kanjang samples resulted in lower weight gain than the normal control group. Interestingly, the visceral fat mass (combined retroperitoneal and epididymal fat) showed no significant differences between the control and the normal control groups. However, leg skeletal muscle mass was reduced in the control group compared to the normal control (Table 1). Among the kanjang treatments, SS-HB was the most effective at reducing body weight gain. Visceral fat contents were lower in the order of positive control = control > SS-MB = SS-FM > SS-HB> SS-LB. All the kanjang treatments, except SS-LB, restored skeletal muscle mass to levels comparable with the normal control group (Table 1). Food efficiency was not significantly different between the control and normal control groups, while all kanjang treatments showed decreased food efficiency relative to the control group. Overall, SS-HB demonstrated the maximum beneficial effects on energy metabolism by simultaneously increasing skeletal muscle mass and reducing visceral fat accumulation (Table 1).

### 3.3. Effects of Kanjang Treatments on Water Homeostasis

Water metabolism was significantly impaired in the scopolamine-injected control group, as indicated by elevated serum levels of renin (54%), angiotensin II (35%), and aldosterone (57%), along with a 75% increase in angiotensin-converting enzyme activity, compared to the normal control group (Table 1). Water intake in the scopolamine-injected control group decreased by approximately 39% compared to the normal control. Water metabolism did not significantly improve in the positive control group. However, all the kanjang treatments prevented increasing the serum levels of renin, angiotensin II, aldosterone concentration, and angiotensin-converting-enzyme activity, thereby improving body water homeostasis (Table 1). Among the kanjang samples, SS-MB and SS-FM showed the most improvement, restoring parameters to levels comparable to the normal control (Table 1).

### 3.4. Effects of Kanjang Treatments on Glucose Metabolism

Fasting serum glucose levels did not differ significantly between the control and normal control groups, while insulin levels were elevated in the control group. All the kanjang treatments except SS-FM reduced serum glucose levels compared to the control. Similarly, serum insulin was lower in all the kanjang groups except SS-MB and SS-FM (Table 2). The homeostatic model assessment for insulin resistance (HOMA-IR) was higher in the control (52%) and positive control (48%) groups versus the normal control. The kanjang treatments reduced HOMA-IR, with the SS-HB group achieving values comparable to normal control (Table 2).

**Figure 1:**
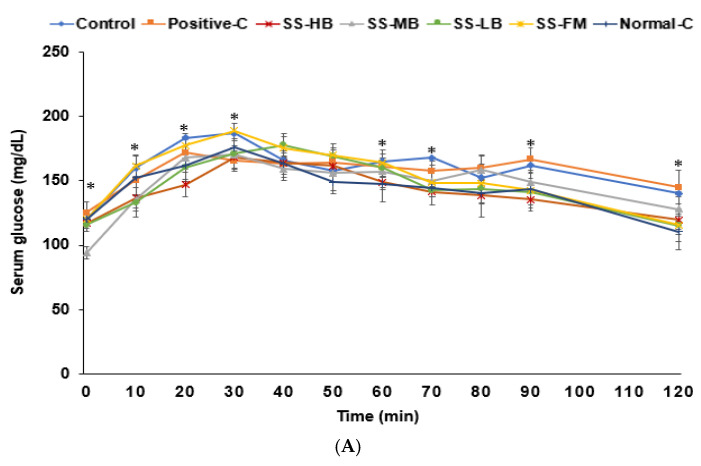

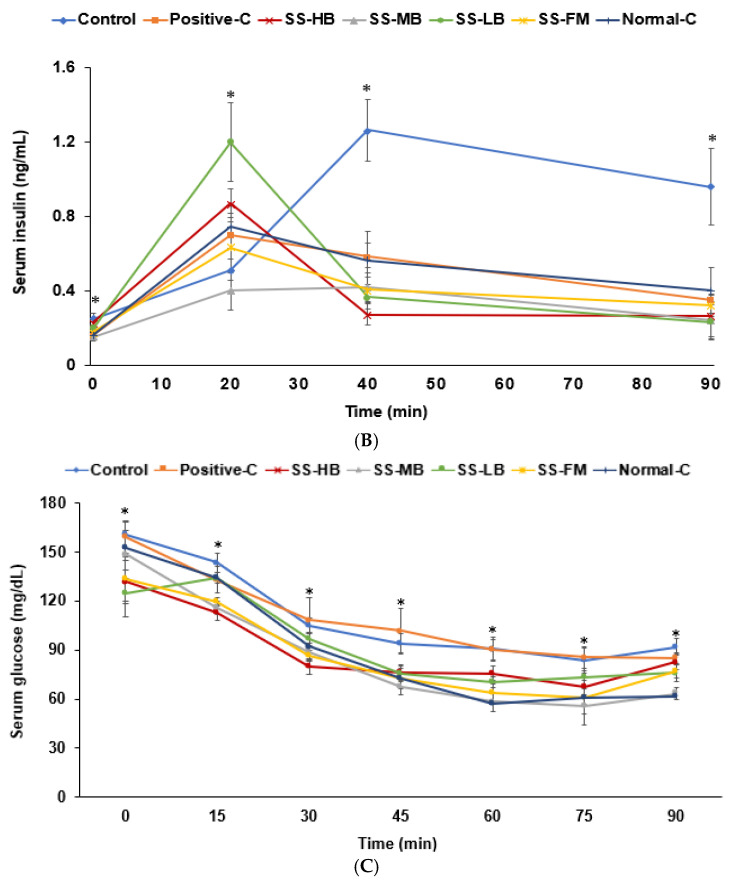
Serum glucose concentrations during the oral glucose tolerance test (OGTT) and intraperitoneal insulin tolerance test (IPITT). (**A**) Changes in serum glucose concentrations after oral intake of 2 g of glucose per kg of body weight (OGTT). (**B**) Changes in serum insulin concentrations during OGTT. (**C**) Changes in serum glucose concentrations after intraperitoneal injection of 1 U of insulin per kg of body weight. Dots and error bars represent the means ± standard deviations (n = 10). * indicates significant differences among the groups at each time point in one-way ANOVA at *p* < 0.05.

Glucose intolerance was evident in the control group in the oral glucose tolerance test (OGTT). Peak glucose levels at 30 min were lower in the SS-HB, SS-MB, SS-LB, and positive control groups compared to the control (Figure 1A). The SS-HB, SS-MB, and SS-LB groups exhibited the lowest serum glucose concentrations, similar to the normal control (Figure 1A). The area under the curve for glucose (AUCG) during both phases of the OGTT was higher in the control group than in the normal control group. All the kanjang treatments except SS-FM reduced the AUC values in the first phase, while all the treatments decreased the AUC in the second phase (Figure S3A).

For the insulin response, the control group showed increased levels until 40 min, followed by a decline, while the kanjang and positive control groups peaked at 20 min. At 20 min, the SS-LB group showed higher insulin than the normal control, while at 40 min, the control group showed higher levels than all groups (Figure 1B). At 90 min, the control group displayed higher insulin levels than the other groups (Figure 1B). The first-phase insulin AUC was highest in the control and SS-LB, with the SS-MB group showing the lowest values. The second-phase AUC was the highest in the control, and it was lower in all kanjang groups than in the control group (Figure S3B). The SS-HB and SS-LB groups showed lower values than those in the normal control group (Figure S3B).

In the IPITT, fasting glucose at 6 h was higher in the control and positive control groups versus the normal control (Figure 1C). Following the insulin injection, glucose levels markedly decreased until 30 min (first phase) and fluctuated between 45 and 90 min (second phase) across all groups. The kanjang treatments decreased serum glucose concentration at 30, 45, and 90 min, with SS-MB reducing glucose to levels similar to the normal control group (Figure 1C). Notably, kanjang intake, but not donepezil (positive control), improved glucose metabolism (Figure 1C and Figure S3C).

### 3.5. Effects of Kanjang Treatments on Serum Markers of Inflammation

Kanjang treatments affected the levels of inflammatory markers. Serum TNF-α and IL-1β concentrations, indicators of inflammation, were elevated in the control group compared to the normal control group. The levels of these markers were not significantly different in the positive control group compared to the control group. Kanjang consumption, except SS-FM, decreased serum TNF-α compared to the control group, though not to the levels observed in the normal control group (Table 2). SS-HB and SS-MB had lower serum IL-1β concentrations than the control group (Table 2).

### 3.6. Effects of Kanjang Treatments on Memory Impairment

**Figure 2:**
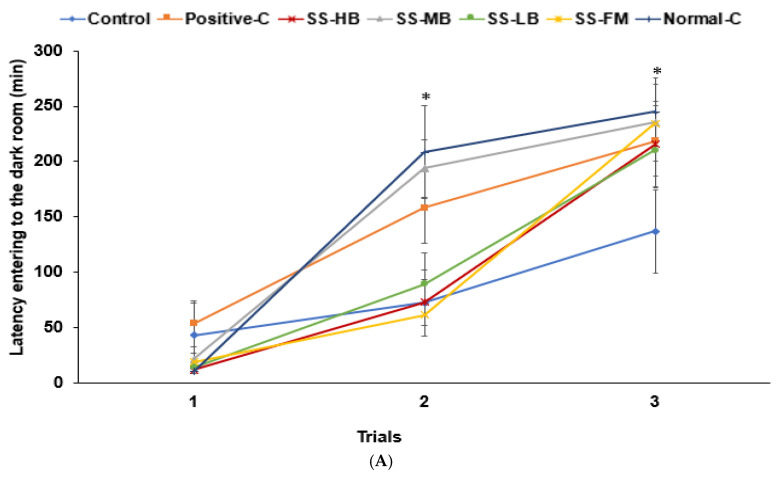

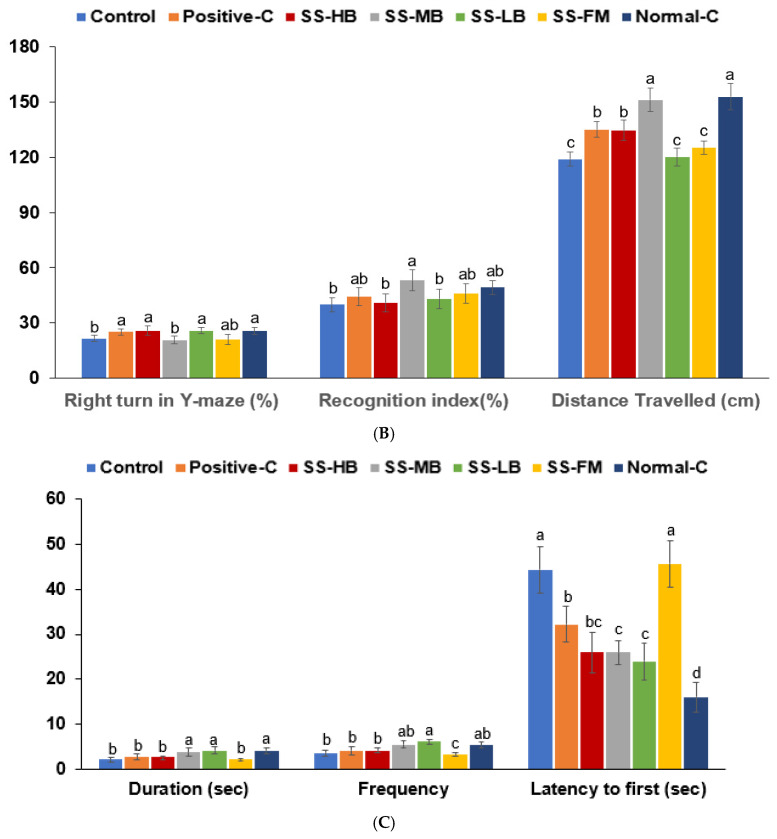

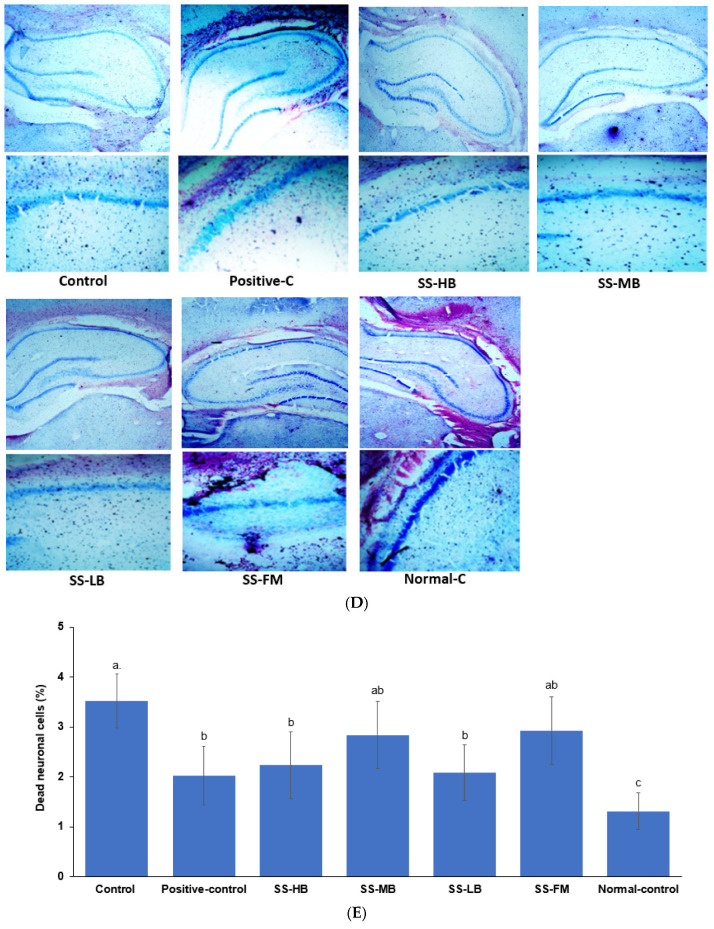
Memory deficits and hippocampal cornu Ammonis (CA1) neuronal cell death in rats after a scopolamine injection. At 50 min after the scopolamine injection, the passive avoidance test, Y-maze, novel object recognition, and Morris water maze test were performed, and hippocampal neuronal cell death was assessed. (**A**) Latency time (s) to enter the dark box at the first, second, and third trials. (**B**) The number of right turns among the total turns in the Y-maze test, novel object recognition index during the novel object recognition test, and distance traveled in locomotor activity measurement. (**C**) Latency time to the first visit, frequency of visiting zone 5, and duration in zone 5 in the water maze test. (**D**) The image of the hippocampal area stained with cresyl violet. Magnification was ×50 in the upper panels and ×100 in the lower panels. (**E**) Neuronal cell death (%) in the hippocampal cornu Ammonis (CA1) stained with cresyl violet. Dots or bars and error bars represent the means ± standard deviations (n = 10). * significantly different among the groups at *p* < 0.05. ^a,b,c^ different superscript letters on the bars indicated a significant difference among the groups by Tukey’s test at *p* < 0.05.

In the passive avoidance test, the rats in the control group entered the dark room quickly, while the rats in the normal control group showed a longer latency. Treatment with kanjang and donepezil (positive control) increased latency time, indicating an improvement in short-term memory (Figure 2A). In the Y-maze test, the control group exhibited fewer correct rotations than the normal control, but SS-HB and SS-LB increased correct rotations to levels comparable with the positive control (Figure 2B). Novel object recognition analysis showed no significant difference between the control and normal control groups in the recognition index, and SS-MB increased this parameter compared to the control (Figure 2B). Locomotive activity was much lower in the control group than in the normal control group. SS-HB increased the activity levels similar to the positive control group, and SS-MB had a similar activity level to the normal control (Figure 2B).

In the Morris water maze test, the duration in zone 5 was lower in the control group than in the normal control and was similar in the SS-MB, SS-LB, and normal control groups (Figure 2C). The frequency of visits to zone 5 was higher in SS-LB than in the control group. The latency to first visit zone 5 was 3-fold higher in the control group than in the normal control. It was lower in the positive control, SS-HB, SS-MB, and SS-LB groups than in the control group, though higher than in the normal control group (Figure 2C). Thus, spatial memory improved in all the kanjang groups, except SS-FM, to levels comparable to or better than the positive control group.

### 3.7. Effects of Kanjang Treatments on Brain Neuronal Cell Death

Neuronal cell death in the hippocampal CA1 region was approximately 2-fold higher in the control group than the normal control. SS-HB and SS-LB significantly reduced neuronal cell death compared to the control group, with effects comparable to the positive control group, although the values were not fully restored to normal control levels (Figure 2D).

The hippocampal glycogen content was significantly lower in the control than in the normal control group, with no significant difference observed between the positive control and control groups (Table 2). All the kanjang treatments increased glycogen content to levels similar to the normal control group. Hippocampal cholesterol was elevated in the control group compared to the normal control, with the kanjang treatments further decreasing these levels. The positive control group showed lower hippocampal cholesterol than the normal control group (Table 2). The hippocampal triglyceride content was higher in the control group than in the normal control group. SS-HB and SS-LB reduced triglyceride content compared to the control, while no significant difference was observed between the positive control and the control groups.

Acetylcholinesterase activity was approximately 3.8-fold higher in the control than in the normal control group (Table 2). All the kanjang treatments reduced this activity, though not to the same extent as observed in the positive control. SS-MB and SS-LB were more effective in reducing acetylcholinesterase activity than SS-HB and SS-FM.

The mRNA expression of *TNF-α* in brain tissue was elevated in the control group relative to the normal control (Table 2). While the positive control did not show significantly reduced *TNF-α* expression, the SS-FM, SS-MB, and SS-LB treatments decreased *TNF-α* levels, and treatment with SS-MB reduced the expression to levels comparable to that of the normal control. Hippocampal *IL-1β* mRNA expression decreased in all treatment groups compared to the control group, with SS-MB showing the biggest reduction (Table 2).

BDNF mRNA expression was approximately 3-fold lower in the control group than the normal control group. All the kanjang treatments and positive control increased *BDNF* mRNA expression compared to the control, with SS-LB and SS-HB showing the most substantial effects, increasing *BDNF* mRNA expression by approximately 2-fold (Table 2).

### 3.8. Effects of Kanjang Treatments on Goblet Cells and Serum SCFA Concentrations

**Figure 3:**
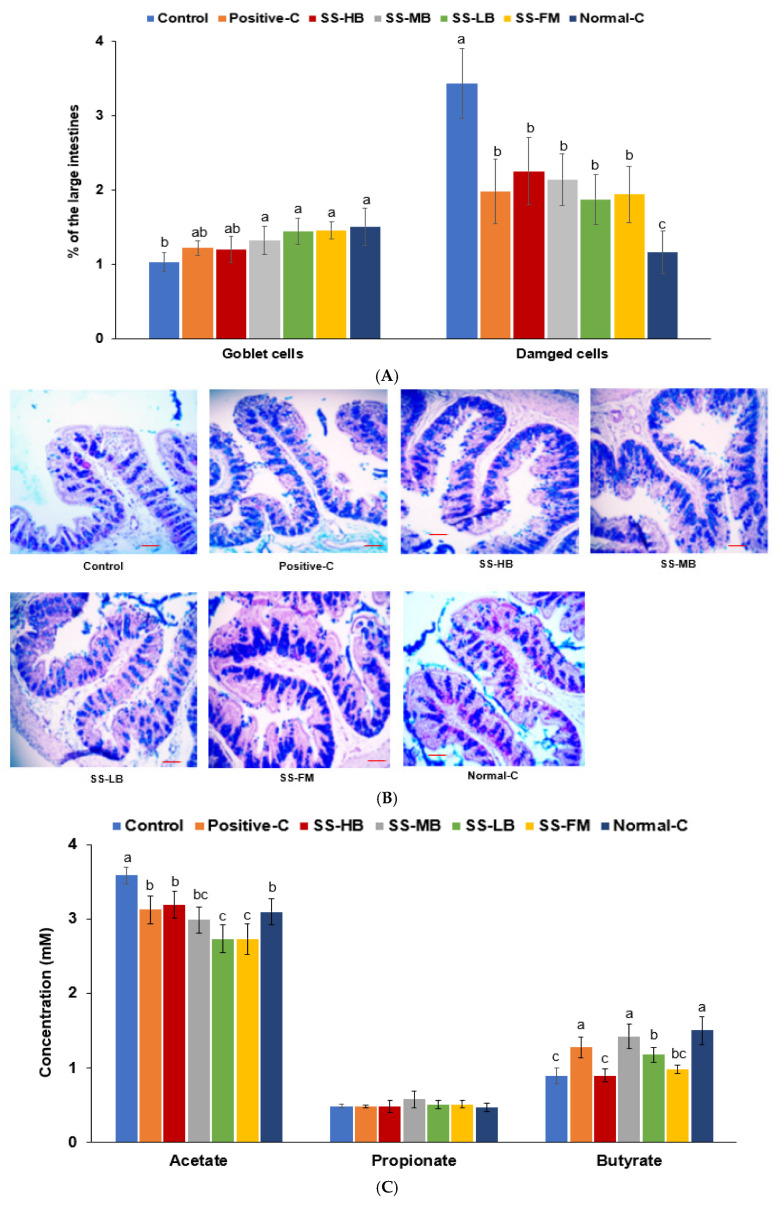
Large intestinal morphology and serum SCFA concentrations after the intervention. (**A**) Damaged cells and goblet cell count. (**B**) Alcian blue–perchloric acid–Schiff (AB-PAS) stain of the intestinal tissues. The violet color indicates mucin. (**C**) Serum short-chain fatty acid (SCFA) levels. The scale bar indicated 100 μm, and the magnification was ×200. Bars and error bars represent the means ± standard deviations (n = 10). ^a,b,c^ different letters on the bars indicate a significant difference among the groups by Tukey’s test at *p* < 0.05.

The goblet cell count was significantly reduced in the control group (1.5-fold lower) compared to the normal control. Goblet cell count increased by approximately 1.5-fold in the positive control and in all the kanjang treatment groups except for the SS-HB group, compared to the control, though it was not fully restored to normal control levels (Figure 3A,B). Mucosal length and crypt depth were lower in the control than in the normal-C group, and kanjang intake increased them (Figure S4A,B).

The serum acetate levels were elevated in the control group compared to the normal control. Both positive control and all kanjang treatments reduced serum acetate levels, with SS-LB and SS-FM decreasing acetate to levels below the normal control (Figure 3C). Serum propionate concentrations showed no significant differences among all the groups. Serum butyrate concentration was higher in the normal control group than in the control group. The serum butyrate levels were significantly higher in the positive control and SS-MB and SS-LB-treated groups compared to the control (Figure 3C). These alterations in goblet cell density and serum SCFA concentrations indicated a disruption of the gut environment, which plays a crucial role in maintaining gut–brain axis integrity.

### 3.9. Effects of Kanjang Treatments on Gut Microbiota and Metagenome Function

**Figure 4:**
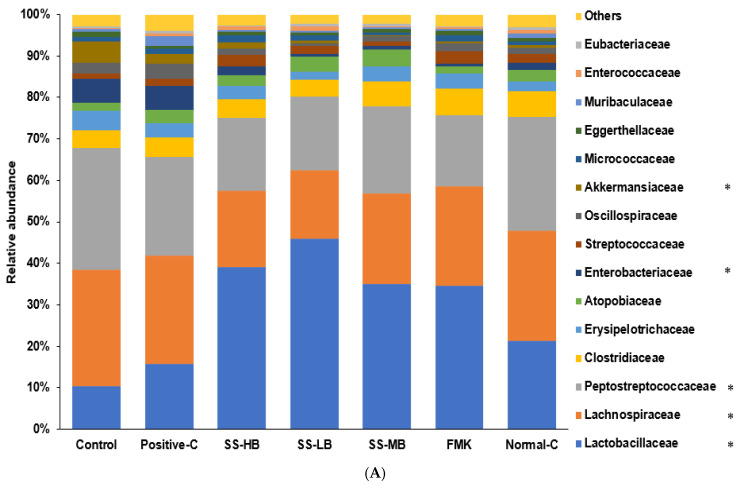

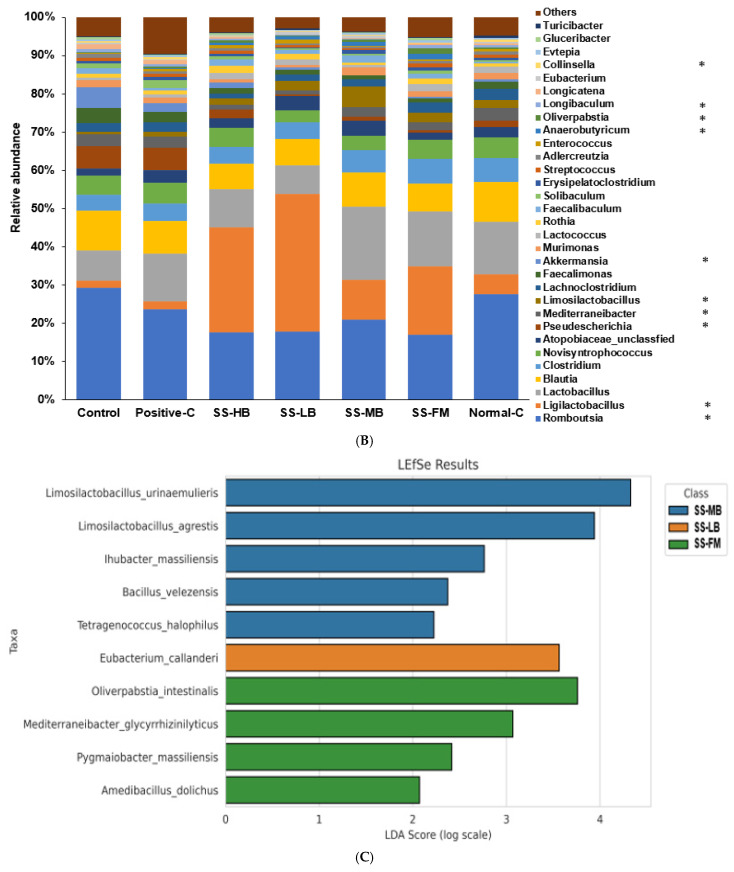

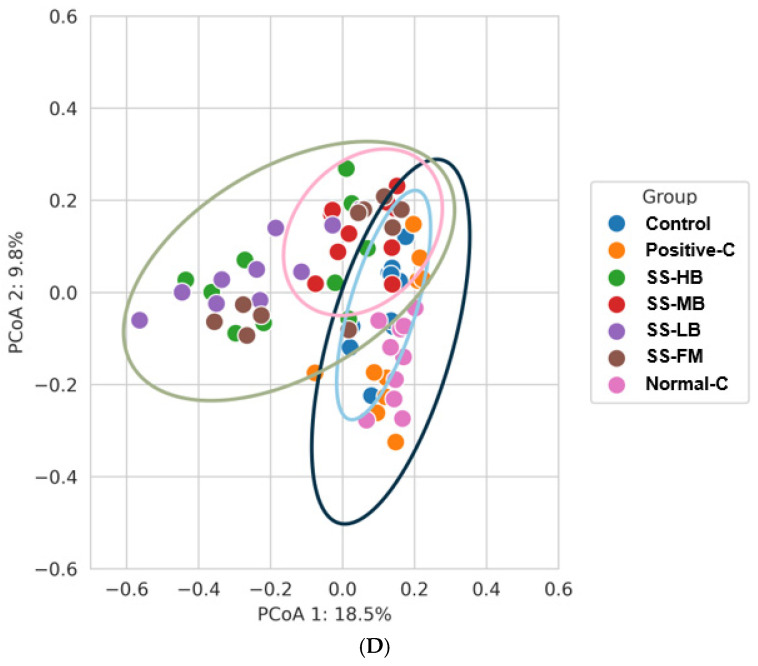
Fecal bacteria composition (**A**). Relative abundance of fecal bacteria from the cecum at the family level (**B**). Relative abundance of fecal bacteria from the cecum at the genus level (**C**). The linear discriminant analysis effect size (LEfSe) analysis (**D**). β-diversity Bars and error bars represent the means ± standard deviations (n = 10). * significantly different among the groups at *p* < 0.05. ^a,b,c^ different letters on the bars indicate a significant difference among the groups by Tukey’s test at *p* < 0.05.

The relative abundance of Lactobacillaceae increased while Lachnospiraceae decreased in all the kanjang-treated groups, with the most pronounced effects in the SS-HB and SS-LB groups (Figure 4A). The Enterobacteriaceae levels were elevated in the control and positive control groups compared to all the kanjang treatment groups (Figure 4A). SS-MB treatment increased the abundance of Lactobacillus more than *Ligilactobacillus*. SS-HB and SS-LB treatments elevated the abundance of *Ligilactobacillus* substantially more than *Lactobacillus* (Figure 4B). *Pseudescherichia* levels were significantly higher in the control and positive control groups compared to the kanjang treatment and normal control groups. *Mediterraneibacter* was less abundant in the SS-HB and SS-LB groups compared to other groups (Figure 4B). *Akkermansia* abundance was higher in the normal control than in the control group, with only SS-HB partially restoring these levels (Figure 4B).

The LEfSE analysis revealed that the SS-MB treatment increased Bacillus-related bacteria, including *Limosilactobacillus urinaemulieris*, *Limosilactobacillus agrestis*, *Ihubacter massiliensis*, and *Bacillus velezensis* (Figure 4C). The SS-LB treatment increased *Eubacterium callanderi*, while SS-FM was associated with higher levels of *Oliverpabstia intestinalis*, *Mediterraneibacter glycyrrhizinilyticus*, and *Pagmaiobacter massiliensis* (Figure 4C). The β-diversity analysis revealed the distinct clustering of fecal bacterial communities in the kanjang-treated group, separating it from both the control and positive control groups. However, no clear separation was observed between the control and positive control groups, indicating their similar microbial compositions (Figure 4D).

Metagenomic functional analysis showed enhanced glycolysis/gluconeogenesis pathways in the normal control group compared to the control (Table 3). All the kanjang treatments except SS-MB enhanced glycolysis/gluconeogenesis and carbohydrate digestion/absorption pathways, suggesting that kanjang intake altered microbial energy metabolism toward fermentative metabolism and increased SCFA production (Table 3). These pathways were most strongly enhanced in the SS-HB and SS-LB groups, exhibiting the highest butanoate metabolism (Table 3).

The peroxisome proliferator-activated receptor (PPAR) and hypoxia-inducible factor (HIF) signaling pathways were more active in the normal control group than in the control and positive control groups (Table 3). All the kanjang treatments increased the activity of these pathways compared to the control. Glutathione metabolism showed no significant differences among the control, positive control, and normal control groups but was enhanced by SS-HB, SS-LB, and SS-FM treatments.

Lipopolysaccharide biosynthesis was more pronounced in the control and positive control groups than in the normal control group, and all the kanjang treatments reduced this pathway. Pro-inflammatory IL-17 signaling was lower in all the kanjang treatment groups, particularly SS-LB, than in the control group (Table 3). Mitophagy was elevated in the control group compared to the normal control group, with all kanjang treatments reducing it. Butanoate metabolism was enhanced in all the kanjang treatment groups compared to the control, while propionate metabolism was reduced in the SS-HB and SS-LB groups (Table 3).

## 4. Discussion

This study examined the effects of four different kanjang varieties on memory function in rats with scopolamine-induced amnesia, revealing significant improvements in cognitive performance alongside beneficial changes in metabolism and gut microbiota composition. While all the kanjang varieties demonstrated protective effects against scopolamine-induced memory impairment, the magnitude and mechanisms of these effects somewhat varied among them.

Among the kanjang varieties evaluated, SS-HB and SS-LB consistently improved cognitive performance across multiple behavioral paradigms, including the Y-maze and Morris water maze tests. In contrast, SS-MB demonstrated selective cognitive enhancement, as evidenced by prolonged latency in the passive avoidance test, a higher novel object recognition index in the novel object recognition test, and an increased traveled distance in locomotor activity. These results suggest that SS-MB exerts targeted effects on recognition memory and exploratory behavior. At the molecular level, the cognitive benefits of SS-MB were supported by the marked suppression of hippocampal pro-inflammatory cytokines (TNF-α and IL-1β), the upregulation of BDNF expression, and reduced AChE activity. Together, these changes imply enhancements in synaptic plasticity, neurotrophic signaling, and cholinergic neurotransmission. While SS-HB and SS-LB also produced favorable outcomes in memory-related behaviors and neuroinflammatory markers, SS-FM showed only modest cognitive effects.

These behavioral outcomes were further corroborated by reduced neuronal cell death in the hippocampal CA1 region and elevated *BDNF* mRNA expression, indicating improved neuronal survival and neuroplasticity. Notably, each kanjang variety exhibited distinct cognitive effects, suggesting that differences in fermentation-derived microbial compositions—such as bacterial species and metabolite profiles—may differentially modulate specific memory domains. These findings are consistent with previous studies, demonstrating that isoflavonoids and *Bacillus*-fermented soybean products improve cognitive function through mechanisms involving *BDNF* upregulation and oxidative stress attenuation in aged or cognitively impaired rodent models [12,13,14]. The high efficacy of SS-HB, which contains a notable abundance of Bacillaceae, aligns with earlier reports showing improvements in memory function and menopausal symptoms in estrogen-deficient rats administered *Bacillus*-fermented soybean products [4]. Similarly, other studies have reported that *Bacillus*-containing fermented foods enhance hippocampal neurogenesis and spatial learning in mouse models of Alzheimer’s disease [15], further reinforcing the neuroprotective role of specific bacterial strains found in SS-HB.

In all kanjang-treated groups, acetylcholinesterase (AChE) activity in the hippocampus was reduced, though not to the extent observed with donepezil in the positive control group. This aligns with previous findings that isoflavone-rich fermented soybeans can inhibit AChE activity in vivo [16,17,18], although less potent than pharmaceutical cholinesterase inhibitors. These results support the potential of kanjang as a dietary strategy for cholinergic modulation, offering complementary cognitive support through nutritional intervention. Collectively, these findings underscore the critical role of microbial ecology and fermentation processes in shaping the neurofunctional potential of traditional fermented soybean products. A comparative overview of the behavioral and molecular outcomes is presented in Table 4, illustrating the differential cognitive efficacy and underlying mechanisms associated with each kanjang variety.

The metabolic improvements observed following kanjang supplementation—particularly enhanced insulin sensitivity, improved glucose tolerance, and reduced fat accumulation—align with previous studies, demonstrating that fermented soybean products ameliorate insulin resistance and metabolic dysfunction in high-fat-diet-induced models [14]. In our study, these effects were most pronounced in the SS-MB group, which showed the greatest reductions in fasting glucose, HOMA-IR, and glucose levels during IPITT, indicating a significant enhancement of systemic insulin sensitivity.

A particularly novel aspect of our findings is the consistent modulation of water metabolism observed in all kanjang-treated groups. Kanjang intake led to increased water consumption and decreased circulating levels of aldosterone, renin, and angiotensin II, suggesting the effective inhibition of the renin–angiotensin–aldosterone system (RAAS). This response was especially marked in SS-MB and SS-FM, both of which exhibited the highest water intake and lowest RAAS activity. As RAAS suppression is associated with reductions in blood pressure, fluid retention, and improved insulin signaling, these findings provide a compelling link between fluid balance regulation and metabolic enhancement. These results are consistent with earlier reports showing that fermented soy consumption attenuates RAAS activity and hypertension [10,19].

In addition to glycemic improvements, several kanjang varieties showed favorable effects on body composition, as shown in a previous study [11]. The SS-HB group demonstrated the most significant reductions in final body weight, visceral fat mass, and food efficiency, suggesting anti-obesity potential. Meanwhile, SS-LB was characterized by elevated insulin levels during OGTT, indicating a possible insulinotropic mechanism that supports glycemic control via increased insulin secretion. These distinct metabolic profiles highlight the role of microbial composition and fermentation characteristics in shaping the functional properties of each kanjang variety. A detailed comparative summary of the metabolic effects—including glucose regulation, insulin sensitivity, RAAS suppression, and body weight control—is presented in Table S2.

Importantly, these metabolic improvements may also contribute to the cognitive enhancements observed in kanjang-treated groups. The interconnection between metabolic health, neuroinflammation, and cognitive function is well established [20,21]. In our study, kanjang intake led to a reduced hippocampal expression of pro-inflammatory cytokines, consistent with previous research demonstrating that fermented soybean extracts inhibit microglial activation and neuroinflammation in LPS-challenged models [22,23]. Notably, SS-MB showed the most robust suppression of hippocampal TNF-α expression, supporting its dual role in improving both metabolic and cognitive function.

Emerging evidence suggests that specific microbial metabolites derived from fermented foods exert anti-inflammatory effects through the inhibition of the NF-κB signaling pathway, a critical mediator of both metabolic and neuroimmune inflammation. Together, our findings support the potential of kanjang varieties—particularly SS-MB, SS-HB, and SS-LB—as multifunctional dietary agents capable of enhancing glucose metabolism, regulating water homeostasis, and attenuating neuroinflammation, with downstream cognitive benefits. The comparative profiles of metabolic outcomes across kanjang types are summarized in Table S2, highlighting their differential efficacy and therapeutic potential.

The alterations in gut microbiota composition induced by the kanjang treatments represent a novel finding extending beyond previous research. Several studies have demonstrated that fermented soy products can modify gut microbiota in different animal models and humans [24,25,26,27,28]. Our study sought to comprehensively analyze the relationship between specific bacterial compositions in kanjang and their effects on host gut microbiota in modifying cognitive function in an amnesic animal model. The increased abundance of *Lactobacillaceae* and *Ligilactobacillus* in the SS-HB and SS-LB groups parallels findings [29,30,31], which reported similar shifts in microbiota following the consumption of fermented soybean foods. Furthermore, our metagenomic functional analysis provides deeper insights into the metabolic consequences of these changes in the microbiota, revealing enhanced glycolysis/gluconeogenesis pathways and butanoate metabolism. Consistent with the metagenome function, the serum butyrate concentration was higher in the SS-MB and SS-LB groups, and a recent study also revealed that soy sauce (kanjang) increased *Agathobacter rectalis* to elevate butyrate production [32]. Elevating serum butyrate levels in the SS-MB and SS-LB groups supports the study that butyrate supplementation improved memory in a mouse model of vascular dementia [33]. Similarly, other studies have shown that butyrate administration enhanced memory formation through increased histone acetylation in the hippocampus, providing a potential mechanism for the cognitive improvements observed in our study [34,35].

The increases in goblet cell count observed in the kanjang-treated groups suggest enhanced gut barrier integrity, consistent with studies which have demonstrated that fermented soybean products strengthen intestinal barrier function and reduce intestinal inflammation in colitis models [36,37]. Our findings extend this work by linking improved gut barrier function to cognitive benefits, supporting the emerging concept that gut permeability plays a crucial role in neurological disorders. Furthermore, kanjang consumption improves memory function through bidirectional gut–brain axis communication. This model aligns with the growing body of evidence linking gut microbiota to cognitive function, potentially through the vagus nervous system [38,39,40]. However, our study is among the first to demonstrate that different varieties of the same traditional fermented food can exert varying effects on this axis, depending on their microbial composition. The superior performance of SS-HB and SS-LB may be attributed to their unique bacterial compositions, which more effectively modulated these pathways. This finding is consistent with recent work, which demonstrated the strain-specific effects of fermented food-derived bacteria on cognitive function and neuroinflammation [18,41].

The estimated sodium intake from the kanjang-supplemented diet was approximately 5.95 mg/day per rat, which, after adjusting for the actual sodium content in fresh kanjang (7%), corresponds to ~19.8 mg/day. Using standard body surface area-based conversion, this equates to approximately 773 mg/day of sodium in a 60 kg adult human, or ~39% of the WHO’s recommended upper intake limit of 2000 mg/day. This sodium level is well within the recognized safety thresholds, supporting the tolerability and translational relevance of the dosage used in this study.

Importantly, no signs of toxicity or adverse effects were observed in the animal model, including no significant differences in food intake among groups. Although the kanjang-treated animals exhibited a lower weight gain, this was attributed to reduced food efficiency rather than reduced appetite or metabolic distress, suggesting a potential metabolic shift rather than harmful effects. These findings, combined with the realistic human-equivalent sodium intake, indicate that dietary supplementation with kanjang is safe and tolerable within the typical sodium range found in traditional diets, particularly when used as a functional substitute for table salt.

### Clinical Implications and Future Directions

These findings have significant implications for developing dietary interventions aimed at preventing cognitive decline. Unlike previous studies that typically examined a single fermented product [42,43,44], our comparative approach across kanjang varieties—each with distinct microbial compositions—underscores the importance of standardizing fermentation processes when developing evidence-based functional foods. The varying cognitive efficacy among different kanjangs also suggests that traditional food processing method, including salt concentrations (typically 8–10%), may provide the empirical knowledge of optimal microbial ecosystems for health benefits. This aligns with a growing interest in traditional fermented foods as sources of next-generation probiotics [45,46]. As the current findings are based on a murine model, future clinical trials will be essential to determine whether the observed improvements in memory, the modulation of neuroinflammation, and gut–brain interaction translate to human cognitive health outcomes.

## 5. Conclusions

**Figure 5:**
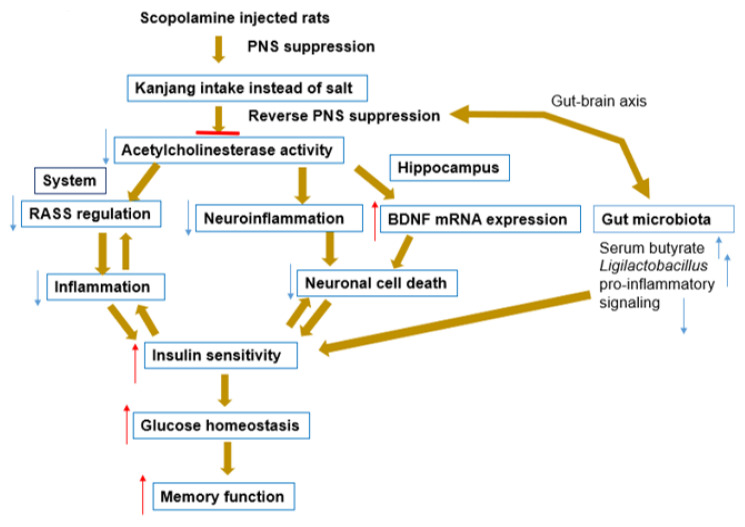
Mechanisms of kanjang intake in reversing scopolamine-induced memory impairment and neuronal cell death. Schematic illustrates the comprehensive mechanisms by which kanjang intake mitigates scopolamine-induced memory impairment and neuronal cell death by parasympathetic nervous system (PSN) suppression with scopolamine injection: (1) reverses PNS suppression, partially by gut microbiota modulation, (2) inhibits acetylcholinesterase activity, (3) increases BDNF mRNA expression and prevents neuronal cell death, (4) modulates the renin–angiotensin–aldosterone system (RASS) and neuroinflammation, and (5) enhances insulin sensitivity. Brown arrows depict interconnected pathways that result in improved memory function and neuronal survival. Small red and blue arrows indicated increase and decrease, respectively. Horizontal red line indicated block of the acetylcholinesterase activity.

This study demonstrates that kanjang consumption, particularly of the SS-HB and SS-LB varieties containing approximately 8–10% sodium, effectively ameliorates scopolamine-induced cognitive dysfunction through a multifaceted mechanistic approach in a murine model. By revealing the interconnected effects on metabolic regulation, neurotrophic signaling, gut microbiota composition, and neuroinflammation, our findings elucidate the complex physiological pathways underlying the cognitive-enhancing properties of kanjang (Figure 5). While high sodium content in fermented foods is typically viewed as unfavorable for health, our results suggest that excessively reducing sodium levels in kanjang may compromise its functional benefits. Traditional fermentation processes require adequate salt concentrations to support the growth of specific microbial communities and the production of bioactive compounds essential for neuroprotection. Our investigation was distinguished by (1) a comparative analysis of kanjang varieties with distinct bacterial compositions, (2) an exploration of microbial metabolites and their role in cognitive function, (3) comprehensive metagenomic functional profiling, and (4) the systems-level integration of metabolic, microbial, and neurological pathways into a unified mechanistic model. The bioactive compounds and microbial signatures identified in kanjang represent a promising foundation for developing targeted nutraceuticals, bridging traditional fermented food knowledge with modern functional food research. Overall, our findings provide not only novel insights into diet-based cognitive interventions but also establish a robust scientific framework for future clinical investigations into the neuroprotective potential of fermented soybean products.

## Figures and Tables

**Table 1:** Visceral fat weight and energy metabolism.

	Control	Positive-C	SS-HB	SS-MB	SS-LB	SS-FM	Normal-C
Final weight (g)	356 ± 5.44 ^a^	362 ± 6.2 ^a^	280 ± 13.3 ^d^	287 ± 7.22 ^cd^	281 ± 13.9 ^d^	299 ± 8.41 ^c^	347 ± 5.61 ^b^
Body weight gain (g)	182 ± 5.44 ^a^	189 ± 6.2 ^a^	96.6 ± 13.3 ^d^	116 ± 7.22 ^c^	110 ± 13.9 ^c^	116 ± 8.41 ^c^	175 ± 5.61 ^b^
Retroperitoneal mass (g)	6.45 ± 0.63 ^a^	6.99 ± 0.64 ^a^	3.08 ± 0.45 ^c^	4.01 ± 0.42 ^b^	2.84 ± 0.32 ^c^	3.9 ± 0.19 ^b^	6.5 ± 0.39 ^a^
Epididymal fat mass	5.14 ± 0.73 ^a^	5.89 ± 0.41 ^a^	1.91 ± 0.43 ^d^	3.22 ± 0.5 ^c^	1.36 ± 0.3 ^e^	2.91 ± 0.33 ^c^	5.43 ± 0.58 ^a^
Visceral fat (% of bw)	3.18 ± 0.32 ^a^	3.53 ± 0.18 ^a^	1.7 ± 0.24 ^c^	2.5 ± 0.19 ^b^	1.47 ± 0.19 ^d^	2.28 ± 0.1 ^b^	2.93 ± 0.24 ^a^
Skeletal muscle (% of bw)	4.39 ± 0.2 ^b^	4.46 ± 0.07 ^b^	4.79 ± 0.1 ^a^	4.73 ± 0.2 ^a^	4.55 ± 0.3 ^b^	4.91 ± 0.15 ^a^	4.76 ± 0.14 ^a^
Food efficiency (%)	0.66 ± 0.03 ^b^	0.74 ± 0.04 ^a^	0.34 ± 0.05 ^d^	0.46 ± 0.03 ^c^	0.38 ± 0.05 ^d^	0.45 ± 0.04 ^c^	0.68 ± 0.04 ^b^
Water intake (mL/day)	17.2 ± 1.27 ^c^	18.6 ± 2 ^c^	27.2 ± 2.27 ^a^	29.7 ± 1.34 ^a^	21.3 ± 1.91 ^b^	29.4 ± 1.34 ^a^	28.3 ± 1.88 ^a^
Serum aldosterone level (ng/mL)	1.24 ± 0.18 ^a^	1.18 ± 0.17 ^a^	0.88 ± 0.11 ^c^	0.73 ± 0.09 ^d^	1.01 ± 0.15 ^b^	0.76 ± 0.09 ^cd^	0.79 ± 0.10 ^d^
Serum renin (pg/mL)	29.4 ± 3.24 ^a^	28.9 ± 3.05 ^a^	22.9 ± 2.23 ^c^	19.4 ± 2.27 ^d^	25.3 ± 2.31 ^b^	19.8 ± 2.32 ^d^	19.1 ± 2.43 ^d^
Serum Angiotensin II (ng/mL)	3.14 ± 0.32 ^a^	3.05 ± 0.21 ^a^	2.49 ± 0.23 ^bc^	2.42 ± 0.32 ^c^	2.75 ± 0.23 ^b^	2.35 ± 0.21 ^c^	2.32 ± 0.21 ^c^
Angiotensin-converting-enzyme activity (U/mL)	2.07 ± 0.27 ^a^	1.98 ± 0.24 ^a^	1.46 ± 0.18 ^c^	1.21 ± 0.17 ^d^	1.67 ± 0.16 ^b^	1.25 ± 0.16 ^d^	1.18 ± 0.17 ^d^

Food efficiency = food intake/body weight × 100. bw, body weight. Values represented means ± standard deviation (n = 10). ^a,b,c,d,e^ Different letters in each variable indicate significant differences in one-way ANOVA at *p* < 0.05.

**Table 2:** Glucose metabolism and lipid profiles.

	Control	Positive-C	SS-HB	SS-MB	SS-LB	SS-FM	Normal-C
Serum glucose (mg/dL)	120 ± 6.53 ^ab^	125 ± 8.42 ^a^	117 ± 1.69 ^b^	94 ± 4.78 ^c^	116 ± 4.85 ^b^	120 ± 3.4 ^ab^	120 ± 3.86 ^ab^
Serum insulin (mg/dL)	0.25 ± 0.03 ^a^	0.22 ± 0.01 ^b^	0.15 ± 0.02 ^b^	0.23 ± 0.02 ^a^	0.18 ± 0.01 ^b^	0.2 ± 0.02 ^ab^	0.16 ± 0.02 ^b^
HOMA-IR	6.61 ± 0.63 ^a^	6.43 ± 0.52 ^a^	3.95 ± 0.34 ^c^	4.95 ± 0.33 ^b^	4.59 ± 0.28 ^b^	5.3 ± 0.31 ^b^	4.35 ± 0.3 ^c^
Serum TNF-α (pg/mL)	0.79 ± 0.08 ^a^	0.73 ± 0.08 ^a^	0.68 ± 0.09 ^b^	0.65 ± 0.08 ^b^	0.68 ± 0.08 ^b^	0.73 ± 0.06 ^a^	0.46 ± 0.05 ^c^
Serum IL-1β (pg/mL)	19.5 ± 1.87 ^a^	18.6 ± 1.63 ^a^	15.3 ± 1.71 ^b^	14.7 ± 1.54 ^b^	17.5 ± 1.58 ^a^	17.2 ± 1.62 ^a^	11.2 ± 1.43 ^c^
Hippocampus							
Glycogen (mg/g)	29.7 ± 1.4 ^b^	30.8 ± 2.1 ^b^	33.4 ± 2.7 ^a^	34.2 ± 2.4 ^a^	33.5 ± 2.5 ^a^	34.8 ± 2.1 ^a^	33.7 ± 2.8 ^a^
Cholesterol (mg/g)	85.8 ± 8.3 ^a^	62.6 ± 8.66 ^c^	69.1 ± 10.1 ^bc^	76.7 ± 9.26 ^b^	71.6 ± 6.16 ^b^	74.9 ± 2.63 ^b^	76.1 ± 8.12 ^b^
Triglyceride (mg/g)	68.1 ± 3.85 ^a^	71.3 ± 3.5 ^a^	61.7 ± 2.62 ^b^	72.5 ± 3.62 ^a^	63.5 ± 4.51 ^b^	69.1 ± 1.93 ^a^	61.1 ± 2.87 ^b^
AChE activity (U/mg protein)	0.85 ± 0.10 ^a^	0.36 ± 0.05 ^d^	0.59 ± 0.07 ^b^	0.43 ± 0.0^6 c^	0.48 ± 0.23 ^c^	0.67 ± 0.08 ^b^	0.22 ± 0.04 ^e^
Relative mRNA of TNF-α (AU)	1 ^a^	0.94 ± 0.08 ^a^	0.95 ± 0.09 ^a^	0.62 ± 0.09 ^c^	0.75 ± 0.08 ^b^	0.88 ± 0.10 ^b^	0.68 ± 0.09 ^c^
Relative mRNA of IL-1β (AU)	1 ^a^	0.78 ± 0.13 ^b^	0.53 ± 0.15 ^c^	0.31 ± 0.09 ^e^	0.64 ± 0.12 ^c^	0.51 ± 0.09 ^d^	0.48 ± 0.04 ^d^
Relative mRNA of BDNF (AU)	1 ^e^	1.53 ± 0.38 ^c^	1.90 ± 0.39 ^bc^	1.47 ± 0.27 ^c^	2.36 ± 0.24 ^b^	1.78 ± 0.33 ^c^	2.94 ± 0.16 ^a^

Values represent means ± standard deviation (n = 10). HOMA-IR, homeostatic model assessment for insulin resistance; HDL, high-density lipoprotein; TG, triglyceride; LDL, low-density lipoprotein; TNF-α, tumor-necrosis factor-α; IL-1β, interleukin-1β; AChE, acetylcholinesterase; BDNF, brain-derived neurotrophic factor; MDA, malondialdehyde; AU, arbitrary unit. ^a,b,c,d,e^ different letters in each variable indicate significant differences in one-way ANOVA at *p* < 0.05.

**Table 3:** Metagenome function of the fecal bacteria from the cecum.

	Control	Positive-C	SS-HB	SS-MB	SS-LB	SS-FM	Normal-C
map00010 glycolysis/gluconeogenesis (86)	2.28 ± 0.03 ^c^	2.31 ± 0.03 ^bc^	2.86 ± 0.09 ^a^	2.66 ± 0.03 ^ab^	3 ± 0.05 ^a^	2.75 ± 0.07 ^a^	2.44 ± 0.02 ^b^
map04973 carbohydrate digestion and absorption (6)	0.02 ± 0.002 ^c^	0.021 ± 0.002 ^c^	0.044 ± 0.005 ^a^	0.025 ± 0.002 ^c^	0.047 ± 0.005 ^a^	0.033 ± 0.005 ^b^	0.022 ± 0.001 ^c^
map03320 PPAR signaling pathway (25)	0.143 ± 0.003 ^c^	0.148 ± 0.006 ^bc^	0.177 ± 0.004 ^a^	0.168 ± 0.006 ^ab^	0.182 ± 0.004 ^a^	0.172 ± 0.003 ^a^	0.151 ± 0.002 ^b^
map04066 HIF-1 signaling pathway (17)	0.528 ± 0.007 ^c^	0.533 ± 0.013 ^bc^	0.616 ± 0.013 ^a^	0.616 ± 0.005 ^a^	0.635 ± 0.006 ^a^	0.612 ± 0.007 ^a^	0.577 ± 0.007 ^b^
map00480 glutathione metabolism (30)	0.327 ± 0.004 ^c^	0.345 ± 0.003 ^c^	0.471 ± 0.007 ^a^	0.395 ± 0.006 ^b^	0.497 ± 0.004 ^a^	0.417 ± 0.008 ^ab^	0.346 ± 0.004 ^c^
map00540 lipopolysaccharide biosynthesis (53)	0.191 ± 0.026 ^a^	0.204 ± 0.025 ^a^	0.115 ± 0.017 ^b^	0.083 ± 0.004 ^c^	0.088 ± 0.006 ^c^	0.089 ± 0.007 ^c^	0.109 ± 0.011 ^b^
map04657 IL-17 signaling pathway (11)	0.057 ± 0.002 ^a^	0.055 ± 0.002 ^a^	0.032 ± 0.003 ^c^	0.041 ± 0.002 ^c^	0.027 ± 0.002 ^d^	0.038 ± 0.003 ^c^	0.052 ± 0.001 ^b^
map04137 mitophagy–animal (15)	0.009 ± 0.001 ^a^	0.009 ± 0.001 ^a^	0.004 ± 0.001 ^c^	0.004 ± 0 ^c^	0.003 ± 0 ^c^	0.003 ± 0.001 ^c^	0.006 ± 0 ^b^
map00650 butanoate metabolism (96)	1.01 ± 0.01 ^c^	1.03 ± 0.01 ^bc^	1.1 ± 0.02 ^a^	1.08 ± 0.01 ^b^	1.13 ± 0.02 ^a^	1.07 ± 0.02 ^b^	1.03 ± 0.01 ^bc^
map00640 propanoate metabolism (84)	1.07 ± 0.02 ^a^	1.11 ± 0.01 ^a^	1.01 ± 0.02 ^b^	1.04 ± 0.01 ^ab^	0.97 ± 0.01 ^b^	1.03 ± 0.02 ^ab^	1.06 ± 0.01 ^b^

Values represent the means ± standard deviations (n = 10). (numbers) indicate the number of reactions involved in the pathways. PPAR, peroxisome proliferator-activated receptor; HIF-1, hypoxia-inducible factor-1; IL-17, interleukin-17. ^a,b,c,d^ different letters on the bars indicate a significant difference among the groups by the Tukey test at *p* < 0.05.

**Table 4:** Integrated summary of neurocognitive and neurochemical effects of different kanjang varieties.

Kanjang Variety	Improved Memory Function	Neuroinflammation by the Hippocampal mRNA Expression	Neuroplasticity (BDNF)	Cholinergic Function (AChE)	Overall Brain Health Effects
SS-HB	Y-maze, locomotive activity, and passive avoidance	↓ TNF-α, ↓↓ IL-1β	↑ BDNF	↓ AChE	Strong multi-dimensional effect
SS-MB	Locomotive activity, novel object recognition, water maze, and passive avoidance	↓↓ TNF-α, ↓↓↓ IL-1β	↑ BDNF	↓↓ AChE	Best behavioral outcome; good mechanistic support
SS-LB	Y-maze, water maze, and passive avoidance	↓↓ TNF-α, ↓↓ IL-1β	↑↑ BDNF	↓↓ AChE	Strong anti-inflammatory and plasticity-related effects
SS-FM	Passive avoidance	Mild anti-inflammatory	↑ BDNF	↓ AChE	Weaker behavioral and molecular effects

Improvements are categorized by hippocampal biochemical markers, inflammation, neuroplasticity, and behavioral cognitive performance. Symbols indicate the direction and relative strength of observed effects compared to the control group. ↑, ↑↑, ↓, ↓↓: Direction/magnitude of improvement. TNF-α, tumor-necrosis factor-α; IL-1β, interleukine-1β; AChE, acetylcholinesterase; BDNF, brain-derived neurotrophic factor.

## Data Availability

The original contributions presented in this study are included in the article/Supplementary Material. Further inquiries can be directed to the corresponding author(s).

## Supplementary Materials

The following supporting information can be downloaded at: https://www.mdpi.com/article/10.3390/nu17101617/s1, Figure S1. Experimental design; Figure S2. Microbiome composition of kanjang samples; Figure S3. Area under the curve (AUC) of serum glucose and insulin concentrations during the oral glucose tolerance (OGTT) and AUC of serum glucose during the intraperitoneal insulin tolerance test (IPITT); Figure S4. Large intestinal morphology after the intervention. (A) Hematoxylin-eosin staining of the intestinal tissues. (B) Mucosal length, crypt width, and crypt depth; Table S1. General characteristics of kanjang samples. Table S2. Integrated summary of the metabolic effects of different kanjang varieties across key domains: energy balance, water regulation, glucose metabolism, and inflammatory status.

## References

[1] Kim S.-H., Ko J., Kwon D.Y. (2023). Jang, Korean fermented soybean product, the result of endeavors of ancients for the best taste of Korean diet. J. Ethn. Foods.

[2] Faraco G., Brea D., Garcia-Bonilla L., Wang G., Racchumi G., Chang H., Buendia I., Santisteban M.M., Segarra S.G., Koizumi K. (2018). Dietary salt promotes neurovascular and cognitive dysfunction through a gut-initiated TH17 response. Nat. Neurosci..

[3] Jeong S.J., Yang H.J., Yang H.G., Ryu M.S., Ha G., Jeong D.Y., Park S. (2023). Inverse association of daily fermented soybean paste (“Jang”) intake with metabolic syndrome risk, especially body fat and hypertension, in men of a large hospital-based cohort. Front. Nutr..

[4] Park S., Zhang T., Yue Y., Jeong S.J., Ryu M.S., Wu X., Yang H.J., Jeong D.Y. (2022). Alleviation of metabolic disturbance by substituting kanjang high in Bacillus for salt through modulation of gut microbiota in estrogen-deficient Rats. Foods.

[5] Zheng Y., Bonfili L., Wei T., Eleuteri A.M. (2023). Understanding the gut-brain axis and Its therapeutic implications for neurodegenerative disorders. Nutrients.

[6] Lu S., Zhao Q., Guan Y., Sun Z., Li W., Guo S., Zhang A. (2024). The communication mechanism of the gut-brain axis and its effect on central nervous system diseases: A systematic review. Biomed. Pharmacother..

[7] Skalny A.V., Aschner M., Gritsenko V.A., Martins A.C., Tizabi Y., Korobeinikova T.V., Paoliello M.M.B., Tinkov A.A. (2024). Modulation of gut microbiota with probiotics as a strategy to counteract endogenous and exogenous neurotoxicity. Adv. Neurotoxicol..

[8] Martin M., Boulaire M., Lucas C., Peltier A., Pourtau L., Gaudout D., Layé S., Pallet V., Joffre C., Dinel A.-L. (2024). Plant extracts and ω-3 improve short-term memory and modulate the microbiota–gut–brain axis in D-galactose model mice. J. Nutr..

[9] Poceviciute I., Brazaityte A., Buisas R., Vengeliene V. (2025). Scopolamine animal model of memory impairment. Behav. Brain Res..

[10] Mun E.G., Sohn H.S., Kim M.S., Cha Y.S. (2017). Antihypertensive effect of Ganjang (traditional Korean soy sauce) on Sprague-Dawley rats. Nutr. Res. Pract..

[11] Lee E.J., Song J., Park C.H., Mun E.G., Wang J., Han A., Park J.E., Cha Y.S. (2023). Soy sauce lowers body weight and fat mass in high-fat diet-induced obese rats. J. Med. Food.

[12] Graham S.E., Clarke S.L., Wu K.-H.H., Kanoni S., Zajac G.J.M., Ramdas S., Surakka I., Ntalla I., Vedantam S., Winkler T.W. (2021). The power of genetic diversity in genome-wide association studies of lipids. Nature.

[13] Ke B., Zhang T., An T., Lu R. (2020). Soy isoflavones ameliorate the cognitive dysfunction of Goto-Kakizaki rats by activating the Nrf2-HO-1 signaling pathway. Aging.

[14] Gopikrishna T., Suresh Kumar H.K., Perumal K., Elangovan E. (2021). Impact of Bacillus in fermented soybean foods on human health. Ann. Microbiol..

[15] Kim B.V., Yoon S., Kim J.E. (2016). A review of fermented foods with beneficial effects on brain and cognitive function. Prev. Nutr. Food Sci..

[16] do Prado F.G., Pagnoncelli M.G.B., de Melo Pereira G.V., Karp S.G., Soccol C.R. (2022). Fermented soy products and their potential health benefits: A review. Microorganisms.

[17] Qiao Y., Zhang K., Zhang Z., Zhang C., Sun Y., Feng Z. (2022). Fermented soybean foods: A review of their functional components, mechanism of action and factors influencing their health benefits. Food Res. Int..

[18] Ryu M.S., Yue Y., Li C., Yang H.-J., Zhang T., Wu X., Jeong D.Y., Park S. (2024). Moderate capsaicin-containing kochujang alleviates memory impairment through the gut-brain axis in rats with scopolamine-induced amnesia. Biomed. Pharmacother..

[19] Zhong B., Mun E.-G., Wang J.-X., Cha Y.-S. (2021). Chinese traditional fermented soy sauce exerts protective effects against high-fat and high-salt diet-induced hypertension in Sprague-Dawley rats by improving adipogenesis and renin-angiotensin-aldosterone system activity. Fermentation.

[20] Zaprovalna O.E., Kolesnikova O.V., Potapenko A.V., Radchenko A.O. (2022). Association between metabolic parameters and cognitive dysfunction in different age groups. Atherosclerosis.

[21] Yulug B., Altay O., Li X., Hanoglu L., Cankaya S., Lam S., Velioglu H.A., Yang H., Coskun E., Idil E. (2023). Combined metabolic activators improve cognitive functions in Alzheimer’s disease patients: A randomized, double-blinded, placebo-controlled phase-II trial. Transl. Neurodegener..

[22] Shalal O.S. (2023). Anti-inflammatory activity of fermented soybean (Glycine max) extract on macrophages by inhibiting cytokines expression. Gene Rep..

[23] Das D., Sarkar S., Borsingh Wann S., Kalita J., Manna P. (2022). Current perspectives on the anti-inflammatory potential of fermented soy foods. Food Res. Int..

[24] Arcusa R., Villaño D., Marhuenda J., Cano M., Cerdà B., Zafrilla P. (2022). Potential role of ginger (Zingiber officinale Roscoe) in the prevention of neurodegenerative diseases. Front. Nutr..

[25] Muniyappan M., Shanmugam S., Park J.H., Han K., Kim I.H. (2023). Effects of fermented soybean meal supplementation on the growth performance and apparent total tract digestibility by modulating the gut microbiome of weaned piglets. Sci. Rep..

[26] Zhang L., Su H., Wang S., Fu Y., Wang M. (2025). Gut microbiota and neurotransmitter regulation: Functional effects of four traditional Chinese fermented soybeans (Sojae Semen praeparatum). Foods.

[27] Song E.-J., Kim M.J., Jung C.H., Chung W.-H., Nam Y.-D., Lim M.Y. (2023). Early response of the gut microbiome and serum metabolites to Cheonggukjang intake in healthy Korean subjects. J. Funct. Foods.

[28] Karbownik M.S., Mokros Ł., Dobielska M., Kowalczyk M., Kowalczyk E. (2022). Association between consumption of fermented food and food-derived prebiotics with cognitive performance, depressive, and anxiety symptoms in psychiatrically healthy medical students under psychological stress: A prospective cohort study. Front. Nutr..

[29] Zhu J.-j., Gao M.-x., Song X.-j., Zhao L., Li Y.-w., Hao Z.-h. (2018). Changes in bacterial diversity and composition in the feces and colon of weaned piglets after feeding fermented soybean meal. J. Med. Microbiol..

[30] Jang S.-E., Kim K.-A., Han M.J., Kim D.-H. (2014). Doenjang, a fermented Korean soybean paste, inhibits lipopolysaccharide production of gut microbiota in mice. J. Med. Food.

[31] Lim H.J., Park I.S., Seo J.W., Ha G., Yang H.J., Jeong D.Y., Kim S.Y., Jung C.H. (2024). Anti-inflammatory effect of Korean soybean sauce (Ganjang) on mice with induced colitis. J. Microbiol. Biotechnol..

[32] Hayashi K., Uchida R., Horiba T., Kawaguchi T., Gomi K., Goto Y. (2024). Soy sauce-like seasoning enhances the growth of Agathobacter rectalis and the production of butyrate, propionate, and lactate. Biosci. Microbiota Food Health.

[33] Fernando W.M.A.D.B., Martins I.J., Morici M., Bharadwaj P., Rainey-Smith S.R., Lim W.L.F., Martins R.N. (2020). Sodium butyrate reduces brain amyloid-β levels and improves cognitive memory performance in an Alzheimer’s disease transgenic mouse model at an early disease stage. J. Alzheimers Dis..

[34] Ziemka-Nalecz M., Jaworska J., Sypecka J., Polowy R., Filipkowski R.K., Zalewska T. (2017). Sodium butyrate, a histone deacetylase inhibitor, exhibits neuroprotective/neurogenic effects in a rat model of neonatal hypoxia-ischemia. Mol. Neurobiol..

[35] Ge X., Zheng M., Hu M., Fang X., Geng D., Liu S., Wang L., Zhang J., Guan L., Zheng P. (2023). Butyrate ameliorates quinolinic acid-induced cognitive decline in obesity models. J. Clin. Investig..

[36] Basson A.R., Ahmed S., Almutairi R., Seo B., Cominelli F. (2021). Regulation of intestinal inflammation by soybean and soy-derived compounds. Foods.

[37] Wu X., Zhang T., Zhang T., Park S. (2024). The impact of gut microbiome enterotypes on ulcerative colitis: Identifying key bacterial species and revealing species co-occurrence networks using machine learning. Gut Microbes.

[38] Tooley K.L. (2020). Effects of the Human gut microbiota on cognitive performance, brain structure, and function: A narrative review. Nutrients.

[39] Park S., Wu X. (2022). Modulation of the gut microbiota in memory impairment and Alzheimer’s disease via the inhibition of the parasympathetic nervous system. Int. J. Mol. Sci..

[40] Han Y., Wang B., Gao H., He C., Hua R., Liang C., Zhang S., Wang Y., Xin S., Xu J. (2022). Vagus nerve and underlying impact on the gut microbiota-brain axis in behavior and neurodegenerative diseases. J. Inflamm. Res..

[41] Balasubramanian R., Schneider E., Gunnigle E., Cotter P.D., Cryan J.F. (2024). Fermented foods: Harnessing their potential to modulate the microbiota-gut-brain axis for mental health. Neurosci. Biobehav. Rev..

[42] Bhatt P.C., Pathak S., Kumar V., Panda B.P. (2018). Attenuation of neurobehavioral and neurochemical abnormalities in an animal model of cognitive deficits of Alzheimer’s disease by fermented soybean nano nutraceutical. Inflammopharmacology.

[43] Lee H.J., Hwang Y.H., Kim D.H. (2018). Lactobacillus plantarum C29-fermented soybean (DW2009) alleviates memory impairment in 5XFAD transgenic mice by regulating microglia activation and gut microbiota composition. Mol. Nutr. Food Res..

[44] Jeong D.Y., Ryu M.S., Yang H.J., Park S. (2021). γ-PGA-rich chungkookjang, short-term fermented soybeans: Prevents memory impairment by modulating brain insulin sensitivity, neuro-inflammation, and the gut-microbiome-brain axis. Foods.

[45] Nithya A., Misra S., Panigrahi C., Dalbhagat C.G., Mishra H.N. (2023). Probiotic potential of fermented foods and their role in non-communicable diseases management: An understanding through recent clinical evidence. Food Chem. Adv..

[46] Valentino V., Magliulo R., Farsi D., Cotter P.D., O’Sullivan O., Ercolini D., De Filippis F. (2024). Fermented foods, their microbiome and its potential in boosting human health. Microb. Biotechnol..

