# Supplementary material for: Beneficial Effects of Traditional Fermented Soybean Sauce (Kanjang) on Memory Function, Body Water, and Glucose Metabolism: Roles of Gut Microbiota and Neuroinflammation
Source: Nutrients. 2025 May 8;17(10):1617. doi: 10.3390/nu17101617 (PMC12113785; doi:10.3390/nu17101617)
Supplement: Supplementary file 1 [file nutrients-17-01617-s001.zip › nutrients-3611762-supplementary.pdf]

Supplementary Figures and Tables

Figure S1. Experimental design

Eight-week-old Sprague-Dawley (SD) male rats were administered scopolamine (2 mg/kg bw) intraperitoneally to suppress the parasympathetic nerve system (PNS) and induce memory impairment. Kanjang samples were categorized into 1) traditionally made kanjang (TMK) with High *Bacillus* (SS-HB), 2) TMK with Medium *Bacillus* (SS-MB), 3) TMK with Low *Bacillus* + High biogenic amine (SS-LB), and 4) SS-FM (factory-made kanjang with Low *Bacillus*). All diets were based on a modified polyphenol-free AIN-93 high-fat diet (HFD) containing 37% carbohydrates, 20% protein, 43% lard, and essential micronutrients. Treatment diets were supplemented with 0.5 % freeze-dried kanjang of a specific variety with cornstarch, casein, corn oil, and salt (NaCl) adjusted to maintain equivalent nutrient composition (43% energy from fat, 4.65 kcal/g). The total experimental period was 8 weeks.

Figure S2. Microbiome composition of kanjang samples

SS-HB, High *Bacillus*; SS-MB, Medium *Bacillus*; SS-LB, Low *Bacillus*; SS-FM, Low *Bacillus*.

Figure S3. Area under the curve (AUC) of serum glucose and insulin concentrations during the oral glucose tolerance (OGTT) and AUC of serum glucose during the intraperitoneal insulin tolerance test (IPITT).

A. AUC of serum glucose during OGTT
B. AUC of serum insulin during OGTT
C. AUC of serum glucose during IPITT

Figure S4. Large intestinal morphology after the intervention

A. Hematoxylin-eosin staining of the large intestinal tissues.

B. Mucosal length, crypt width, and crypt depth. In the large intestinal tissues

The red bar indicates the scale bar (100 um). Magnification was X100.

Fig. S1

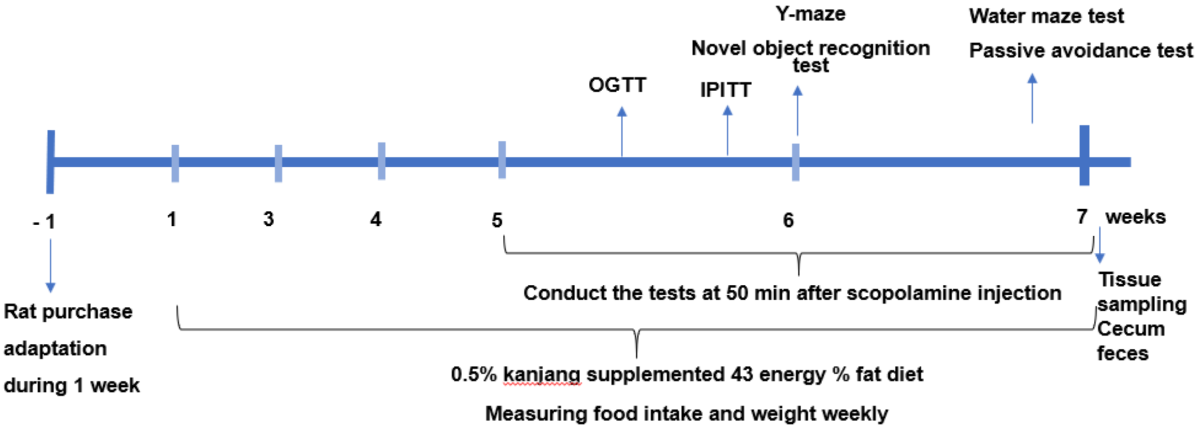
Y-maze
Novel object recognition test
Water maze test
Passive avoidance test
OGTT
IPITT
- 1
1
3
4
5
6
7 weeks
Rat purchase
adaptation
during 1 week
Conduct the tests at 50 min after scopolamine injection
Tissue sampling
Cecum feces
0.5% kanjang supplemented 43 energy % fat diet
Measuring food intake and weight weekly

Fig. S2

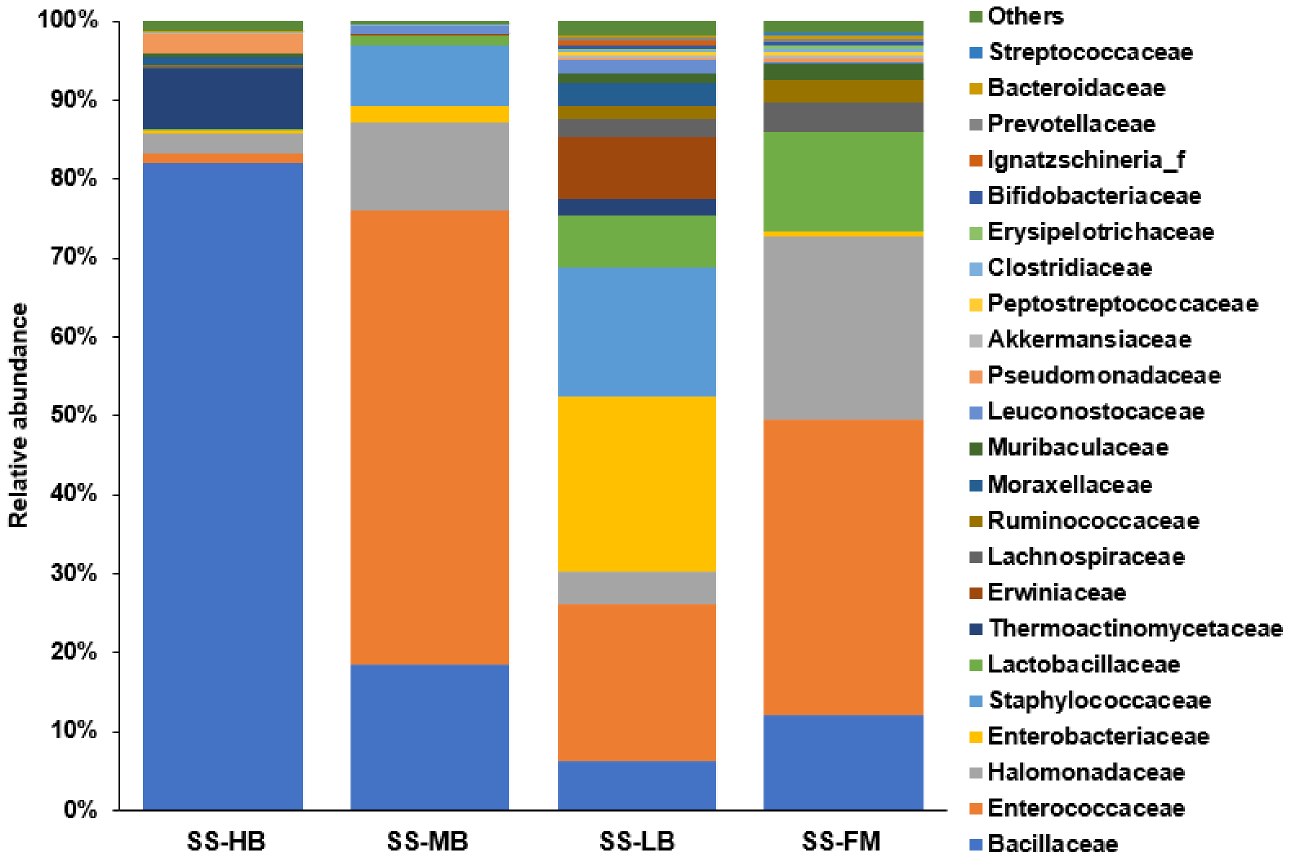
Relative abundance
100%
90%
80%
70%
60%
50%
40%
30%
20%
10%
0%
SS-HB
SS-MB
SS-LB
SS-FM
Others
Streptococcaceae
Bacteroidaceae
Prevotellaceae
Ignatzschineria_f
Bifidobacteriaceae
Erysipelotrichaceae
Clostridiaceae
Peptostreptococcaceae
Akkermansiaceae
Pseudomonadaceae
Leuconostocaceae
Muribaculaceae
Moraxellaceae
Ruminococcaceae
Lachnospiraceae
Erwiniaceae
Thermoactinomycetaceae
Lactobacillaceae
Staphylococcaceae
Enterobacteriaceae
Halomonadaceae
Enterococcaceae
Bacillaceae

Fig. S3A

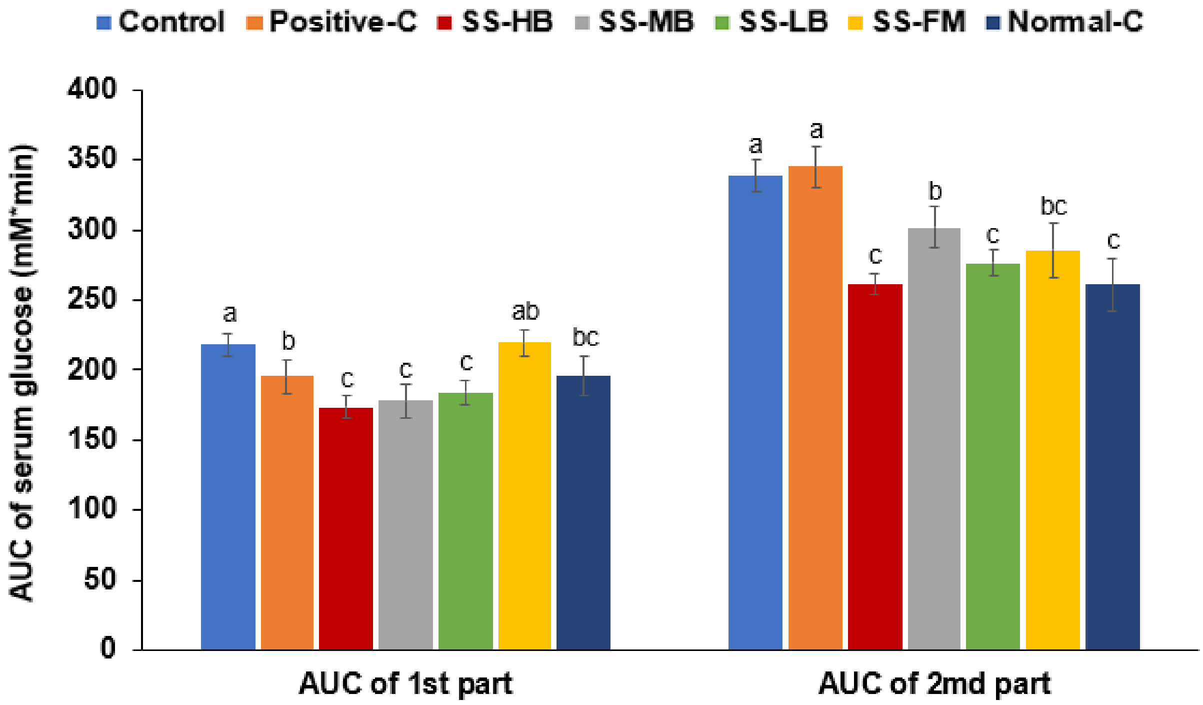

Fig. S3B

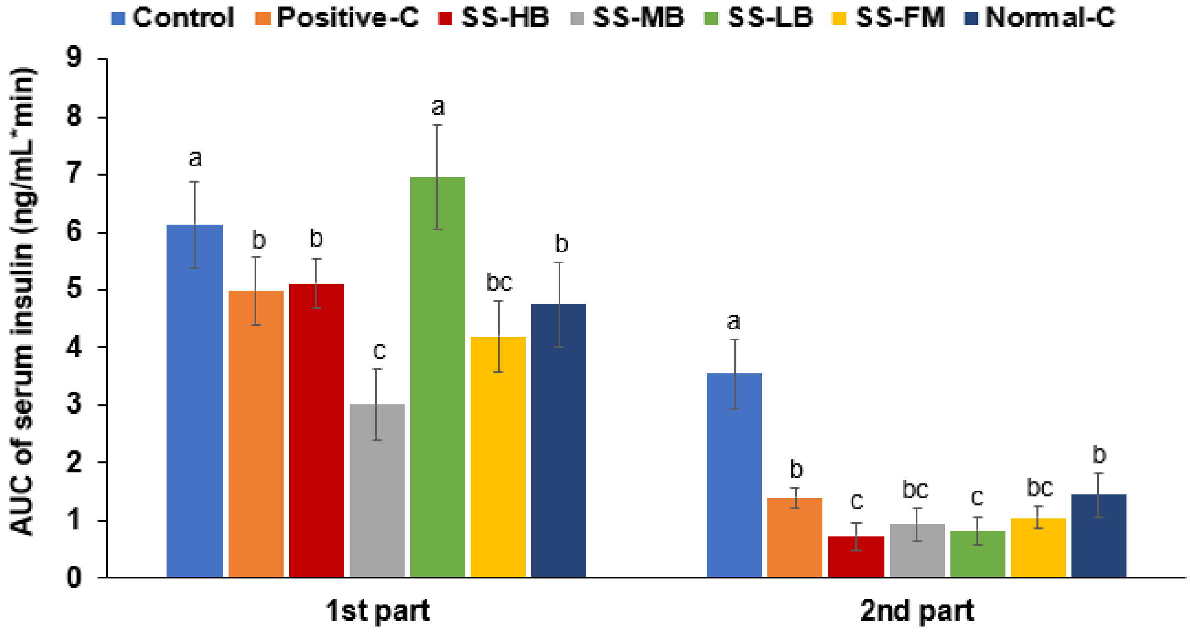

Fig. S3C

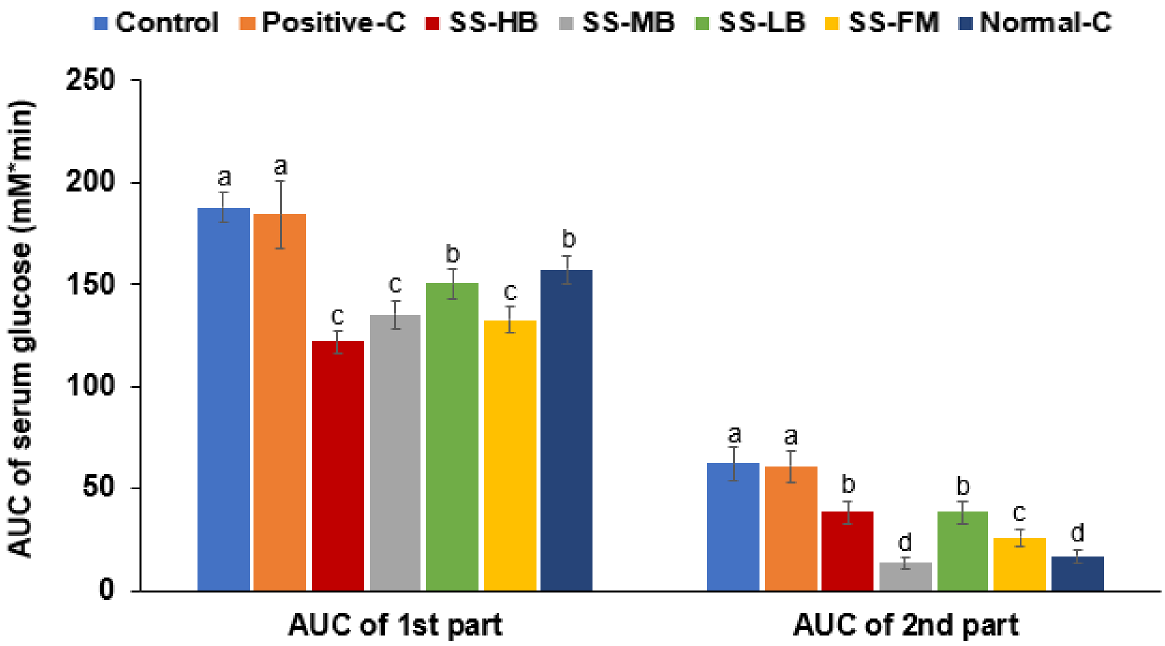

Control
Positive-C
SS-HB
SS-MB
SS-LB
SS-FM
Normal-C
AUC of serum glucose (mM*min)
250
200
150
100
50
0
a
a
c
c
b
c
b
a
a
b
d
b
c
d
AUC of 1st part
AUC of 2nd part

34

Fig. S4A

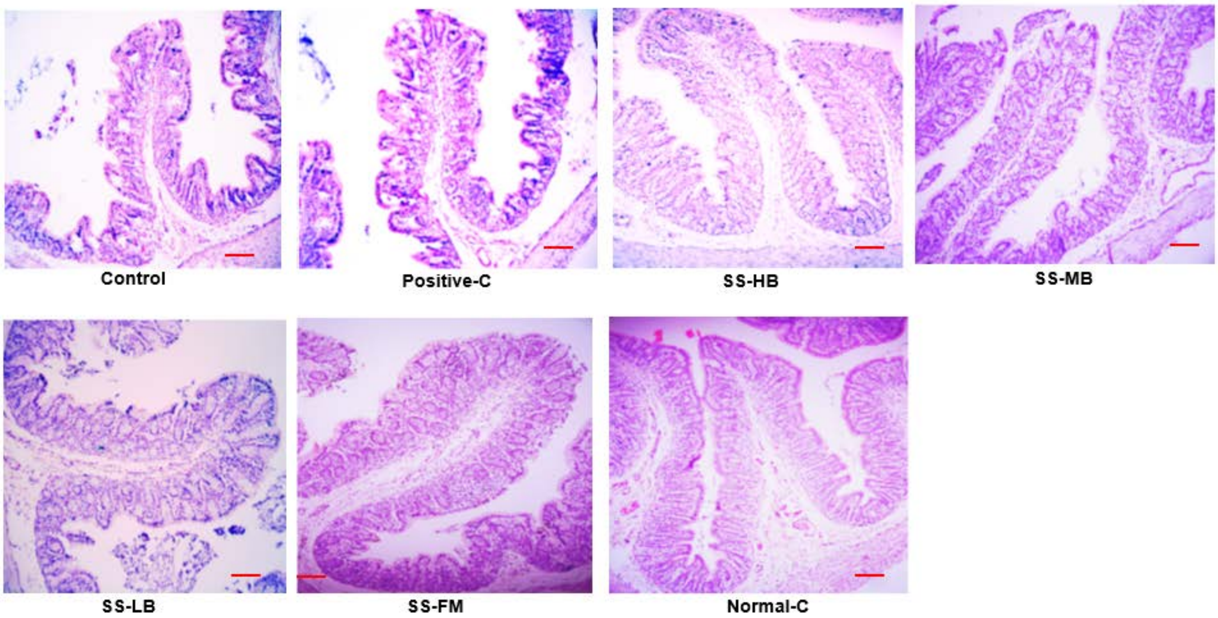

Fig. S4B

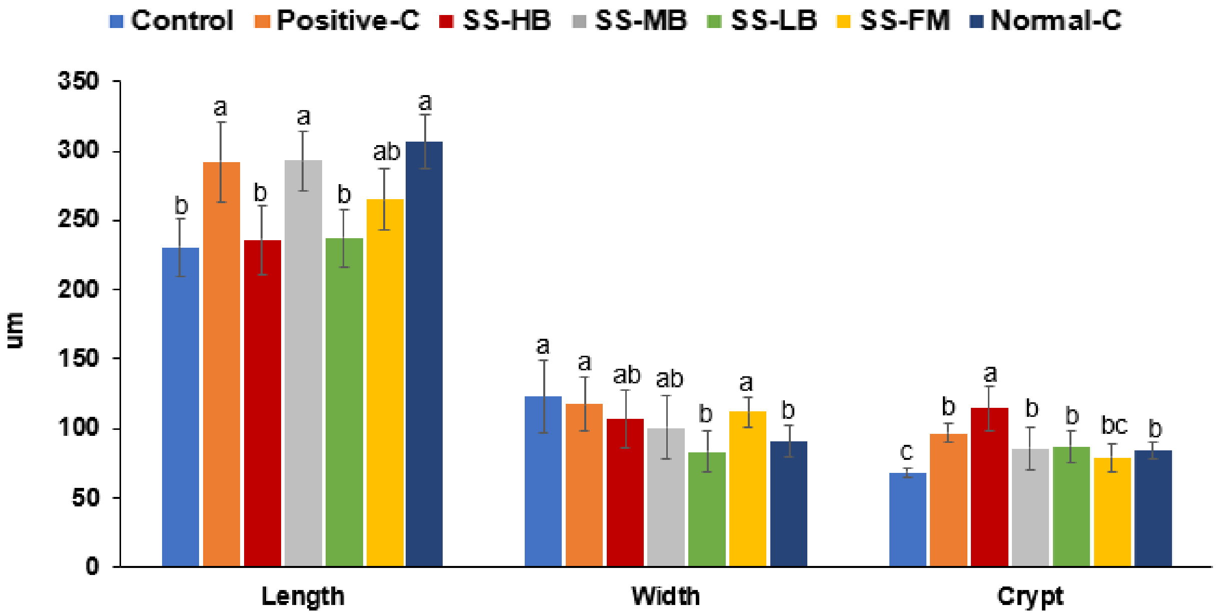

Table S1. General characteristics of kanjang samples

Group	Water (%)	Biogenic amines(mg/kg)		Na (wt%)	Live bacteria (μg/kg)
		Histamine	Tyramine		
SS-HB	68.45	0±0	54.5±1.36	8.59 ± 0.07	1.3×10^{5} ± 0.0004
SS-MB	71.41	484±45.9	854±60.6	5.56 ± 0.06	1.0×10^{7}±0.00003
SS-LB	62.51	420±44	1879±21.3	9.39 ± 0.08	4.3×10^{5}±0.0006
SS-FM	75.21	19.2±2.72	49.4±8.23	6.08 ± 0.07	1.0×10^{3}±0.003

SS-HB, TMK with high contents of *Bacillus* spp.
SS-MB, TMK with medium contents of *Bacillus* spp.
SS-LB, TMK with low contents of *Bacillus* spp.
SS-FM, factory-made low contents of *Bacillus* spp.
Values represented means ± standard deviation (n=5).

Table S2. Integrated summary of the metabolic effects of different kanjang varieties across key domains: energy balance, water regulation, glucose metabolism, and inflammatory status.

Kanjang variety	Body weight & fat mass	Energy efficiency	Water balance & RAAS	Glucose control (Fasting, OGTT)	Insulin sensitivity (HOMA-IR, IPITT)	Overall metabolic effect
SS-HB	↓↓↓ Weight, ↓ Fat mass	↓↓↓ Efficiency	↑↑ Water Intake, ↓ RAAS	↓ Glucose, ↓ AUC in OGTT	↓↓ HOMA-IR, normal insulin sensitivity by IPITT	Strong anti-obesity Moderate insulin sensitizer
SS-MB	↓↓ Weight, Moderate Fat ↓	↓↓ Efficiency	↑↑↑ Water Intake, ↓↓ RAAS	↓↓↓ Glucose (best), ↓ AUC in OGTT	↓↓↓ HOMA-IR, ↑ insulin sensitivity by IPITT	Strong insulin sensitizer Decrease RAAS Water balance
SS-LB	↓↓↓ Weight, ↓↓↓ Fat Mass	↓↓↓ Efficiency	↑ Water Intake, ↓ RAAS	Mild ↓ Glucose, ↑ Insulin (OGTT)	↓ HOMA-IR, moderate insulin sensitivity by IPITT	Insulinogenic & anti-obesity
SS-FM	↓ Weight, Moderate Fat ↓	↓↓ Efficiency	↑↑ Water Intake, ↓ RAAS	Moderate ↓ Glucose, ↓ AUC in OGTT	↓ HOMA-IR, mild insulin sensitivity by IPITT	Decreased RAAS Water balance

↑, ↑↑, ↓↓: Direction/magnitude of improvement. RAAS, renin-angiotensin-aldosterone system; AUC in OGTT, area under the curve of oral glucose tolerance test; HOMA-IR, IPITT, intraperitoneal insulin tolerance test.
